# Identification of species and materia medica within *Saussurea* subg. *Amphilaena* based on DNA barcodes

**DOI:** 10.7717/peerj.6357

**Published:** 2019-02-01

**Authors:** Jie Chen, Yong-Bao Zhao, Yu-Jin Wang, Xiao-Gang Li

**Affiliations:** State Key Laboratory of Grassland Agro-Ecosystem, School of Life Sciences, Lanzhou University, Lanzhou, Gansu, China

**Keywords:** *Saussurea* subg. *Amphilaenais*, Medical plant, Taxonomic problem, DNA barcoding, Substitute

## Abstract

*Saussurea* is one of the most species-rich genera in the family Asteraceae, where some have a complex evolutionary history, including radiation and convergent evolution, and the identification of these species is notoriously difficult. This genus contains many plants with medical uses, and thus an objective identification method is urgently needed. *Saussurea* subg. *Amphilaena* is one of the four subgenera of *Saussurea* and it is particularly rich in medical resources, where 15/39 species are used in medicine. To test the application of DNA barcodes in this subgenus, five candidates were sequenced and analyzed using 131 individuals representing 15 medical plants and four additional species from this subgenus. Our results suggested that internal transcribed spacer (ITS) + *rbc*L or ITS + *rbc*L + *psb*A-*trn*H could distinguish all of the species, while the ITS alone could identify all of the 15 medical plants. However, the species identification rates based on plastid barcodes were low, i.e., 0% to 36% when analyzed individually, and 63% when all four loci were combined. Thus, we recommend using ITS + *rbc*L as the DNA barcode for *S.* subg. *Amphilaena* or the ITS alone for medical plants. Possible taxonomic problems and substitutes for medicinal plant materials are also discussed.

## Introduction

*Saussurea* is one of the most species-rich genera in Asteraceae and the taxonomic identification of these species is notoriously difficult (Lipschitz, 1979). Recent radiation, widespread hybridization, and convergent evolution have combined to make the delimitation of these species extremely complicated (Wang et al., 2009). Among the 289 recognized species in the “Flora of China” (FOC), many are very challenging to differentiate, with one or several morphologically similar species (Shi & Raab-Straube, 2011). For example, about nine current widely accepted species are suspected to be conspecific with *S. taraxacifolia* (Chen, 2015). Since the publication of FOC, the newly described species have totaled more than 60 species (Chen, 2015; Wang et al., 2014; Xu, Hao & Xia, 2014; Chen & Wang, 2018), with an average of 10 species every year, which is a far higher number than that of other genera. These new species have mostly been separated from the known species and at least 10 of them bear the prefix “pseudo” to indicate their similarity in terms of morphology (Chen, 2014; Chen & Yuan, 2015; Wang et al., 2014).

**Figure 1:**
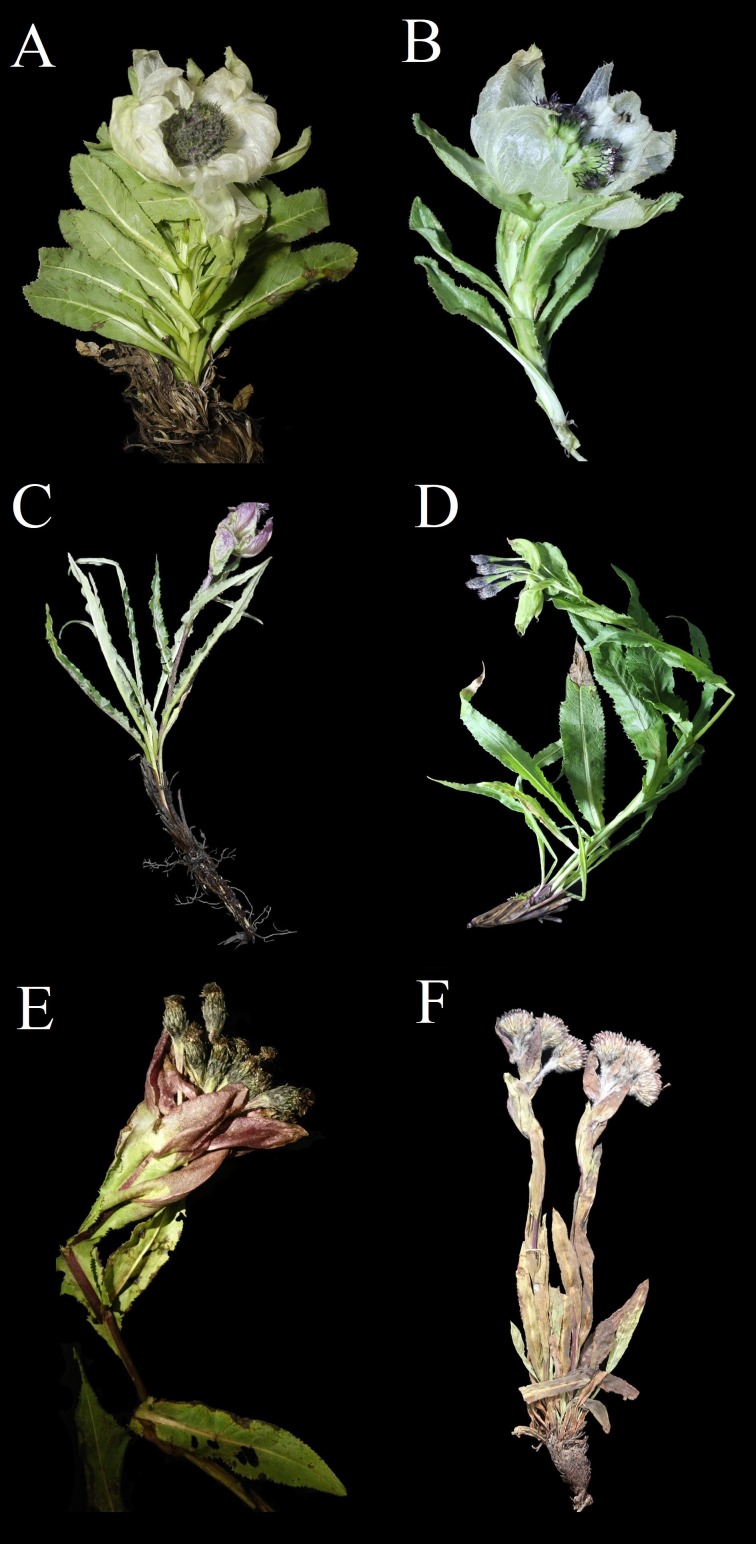
Photographs of six species sampled in the study. (A) *S. bogedaensis*, WYJ201607018. (B) *S. involucrata*, WYJ201607025. (C) *S. pubifolia*, WYJ201607272. (D) *S. luae*, WYJ201607286. (E) *S. globosa*, WYJ201607422. (F) *S. erubescens*, sn110814017. **

This taxonomic problem particularly affects *S.* subg. *Amphilaena*, which is one of the four subgenera of *Saussurea*, where these species are defined mainly based on the self-transparent and colorful bract that subtends the synflorescence (Fig. 1) (Lipschitz, 1979; Raab-Straube, 2017). This character is a well-known adaptation to high altitudes and it occurs in a number of angiosperm genera from different families (Omori, Takayama & Fls, 2000). Within *S.* subg. *Amphilaena*, it has also been documented that this character was derived multiple times and some of the species showing very high similarity, such as *S. involucrata* and *S. obvolata*, are actually distantly related according to molecular phylogeny (Wang et al., 2009). In addition, this subgenus is considered to be a result of a recent radiation in the Qinghai–Tibet Plateau where 35 of the total number of 38 species have been recorded (Raab-Straube, 2017). This type of process usually produces many closely related species where one species might resemble several other species, thereby yielding a number of complexes (Simões et al., 2016).

Complex taxonomy undoubtedly causes problems with identification, and among the 38 species recognized in the latest monograph, at least 13 species are widely misidentified. For example, *S. orgaadayi* was long misidentified as *S. involucrata* (Smirnov, 2004), although both species were described many years ago and the latter is one of the most famous plants in China because of its beauty and usage in traditional Chinese medicine (Chik et al., 2015). In addition, eight species within the *S. obvallata* complex have been recognized as single species since the establishment of *S. obvallata* (Raab-Straube, 2017).

Evidently, misidentification can lead to a misunderstanding of biodiversity. In some cases, these errors can even be deadly harmful for humans given that many *Saussurea* species are used in medicine (Chik et al., 2015; Li, Zhu & Cai, 2000; Yang et al., 2005). In addition to *S. involucrata*, 14 other species have been formally recorded as medically useful in *S.* subg. *Amphilaena* (Table 1) (Cao et al., 2016; Chen, Pei & Zhao, 2010; Jiang, Luo & Xu, 2010; Li, 1999). However, the authentication of species is time-consuming and it requires a specialist taxonomist in most cases. Moreover, some species are found only in areas that are difficult to access, possibly because of their excessive consumption. For example, *S. involucrata* is currently listed as second-class protected plants due to over-exploitation (Fu & Jin, 1992), while *S. wettsteiniana* and *S. velutina* are both endemic to a few mountains in Sichuan, China, and they are difficult to obtain due to their restricted distributions (Shi & Raab-Straube, 2011). Thus, possible substitutes for these species are urgently needed to be ascertained.

**Table 1:** List of medicinal plants within *Saussurea subg. Amphilaena.*

Species	Reference
*S. involuvcrata*	Chen, Pei & Zhao (2010) and Chik et al. (2015)
*S. globosa*	Cao et al. (2016) and Li (1999)
*S. wettsteiniana*	Jiang, Luo & Xu (2010)
*S. polycolea*	Jiang, Luo & Xu (2010) and Li (1999)
*S. uniflora*	Jiang, Luo & Xu (2010) and Li (1999)
*S. velutina*	Jiang, Luo & Xu (2010)
*S. phaeantha*	Cao et al. (2016) and Li (1999)
*S. orgaadayi*	Shi & Raab-Straube (2011)
*S. tangutica*	Cao et al. (2016) and Li, Zhu & Cai (2000)
*S. bracteata*	Li (1999)
*S. erubescens*	Cao et al. (2016) and Li (1999)
*S. nigrescens*	Cao et al. (2016) and Li (1999)
*S. iodostegia*	Cao et al. (2016) and Li (1999)
*S. glandulosissima*	Cao et al. (2016), Li (1999) and Yang et al. (2005)
*S. sikkimensis*	Cao et al. (2016), Li (1999) and Yang et al. (2005)

DNA barcoding is a rapid and reliable technique for identifying species based on variations in the sequence of short standard DNA regions. Phylogenetic studies based on these fragments can also help to identify substitute plants. However, the selection of the fragments used for DNA barcoding is a controversial problem. The Plant Working Group of the Consortium for the Barcode of Life (CBOL) proposed using a combination of *rbc*L and *mat*K as a “core barcode” for identifying land plants (Hollingsworth et al., 2009). Subsequently, *trn*H-*psb*A and the nuclear ribosomal internal transcribed spacer (ITS) were proposed as supplementary barcodes for land plants (Kress et al., 2005; Li et al., 2011). In addition, *trn*K was found to outperform *mat*K in some studies (Cao et al., 2010; Müller & Borsch, 2005).

Previously, the sequences used in DNA barcodes for *Saussurea* species have been rather limited and only five species have been reported with DNA sequences. Among these species, none have been reported more than two populations, which is obviously insufficient for DNA barcode studies (Wang et al., 2009). Thus, in this study, we performed extensive investigations in the field, and we sequenced five DNA barcode candidates in chloroplasts (*mat*K, *trn*H-*psb*A, *trn*K, and *rbc*L) and the nuclear ITS. Our main aims were: (i) to evaluate the application of these DNA barcodes in *S.* subg*. Amphilaena*; (ii) to develop an objective method for identifying medically important *Saussurea* species; and (iii) to explore the possible taxonomic problems and potential substitutes for some rare herbs.

## Materials and Methods

### Taxon sampling

In total, 20 species were sampled in the present study, including 18 from the 38 species recognized in the latest monograph on *S.* subg*. Amphilaena* (Raab-Straube, 2017), one recently published species, *S. bogedaensis* (Chen & Wang, 2018), and a *Jurinea* species, which was selected as an outgroup according to a previous study (Wang et al., 2009). Photos of some species are presented in Fig. 1. Our sample focus on medical resources and 15 species formally recorded in the medical literature were included in the analyses (Table 1). For most of the species in the ingroup, we collected from two or more populations, with more than three individuals from each population. In total, we collected 132 individuals and their details are listed in Table 2.

**Table 2:** The name, locality, voucher and GenBank accession number for the samples used in this study.

Species	Locality (All from China)	Voucher/Individual	Latitude (°)	Longitude (°)	Altitude (m)	GenBank accession number (ITS, *mat*K, *rbc*L, *trn*K, *trn*H-*psb*A)				
*S. bogedaensis*	Qitai, Xinjiang	WYJ201607018b, 140	43.45321	89.55213	3,471	MH003705	MH070617	MH070870	MH070996	MH070743
*S. bogedaensis*	Qitai, Xinjiang	WYJ201607018a, 167	43.45321	89.55213	3,471	MH003706	MH070618	MH070871	MH070997	MH070744
*S. bogedaensis*	Qitai, Xinjiang	WYJ201607018, 378	43.45321	89.55213	3,471	MH003707	MH070619	MH070872	MH070998	MH070745
*S. bogedaensis*	Qitai, Xinjiang	WYJ201308006, 38	43.44370	89.58167	3,386	MH003708	MH070620	MH070873	MH070999	MH070746
*S. bogedaensis*	Qitai, Xinjiang	WYJ201308006, 39	43.44370	89.58167	3,386	MH003709	MH070621	MH070874	MH071000	MH070747
*S. bogedaensis*	Qitai, Xinjiang	WYJ201308006, 40	43.44370	89.58167	3,386	MH003710	MH070622	MH070875	MH071001	MH070748
*S. bracteata*	Qumalai, Qinghai	WYJ201207537, 114	34.84716	94.94569	4,621	MH003711	MH070623	MH070876	MH071002	MH070749
*S. bracteata*	Cuomei, Xizang	WYJ201607213, 151	28.51474	91.45611	4,934	MH003712	MH070624	MH070877	MH071003	MH070750
*S. bracteata*	Cuomei, Xizang	WYJ201607213, 153	28.51474	91.45611	4,934	MH003713	MH070625	MH070878	MH071004	MH070751
*S. bracteata*	Yushu, Qinghai	WYJ201607043, 160	35.05681	93.01225	4,644	MH003714	MH070626	MH070879	MH071005	MH070752
*S. bracteata*	Yushu, Qinghai	WYJ201607043, 161	35.05681	93.01225	4,644	MH003715	MH070627	MH070880	MH071006	MH070753
*S. bracteata*	Yushu, Qinghai	WYJ201607043, 162	35.05681	93.01225	4,644	MH003716	MH070628	MH070881	MH071007	MH070754
*S. bracteata*	Jilong, Xizang	WYJ201607099, 173	28.93494	85.39376	5,108	MH003717	MH070629	MH070882	MH071008	MH070755
*S. bracteata*	Jilong, Xizang	WYJ201607099, 174	28.93494	85.39376	5,108	MH003718	MH070630	MH070883	MH071009	MH070756
*S. bracteata*	Jilong, Xizang	WYJ201607099, 175	28.93494	85.39376	5,108	MH003719	MH070631	MH070884	MH071010	MH070757
*S. bracteata*	Geermu, Qinghai	WYJ201607053f, 204	32.98834	91.98589	5,120	MH003720	MH070632	MH070885	MH071011	MH070758
*S. bracteata*	Geermu, Qinghai	WYJ201607041, 248	35.51127	93.72552	4,525	MH003721	MH070633	MH070886	MH071012	MH070759
*S. bracteata*	Geermu, Qinghai	WYJ201607041, 249	35.51127	93.72552	4,525	MH003722	MH070634	MH070887	MH071013	MH070760
*S. erubescens*	Luqu, Gansu	sn110814017, 123	34.59103	102.48699	3,345	MH003723	MH070635	MH070888	MH071014	MH070761
*S. erubescens*	Luqu, Gansu	sn110814018, 124	34.59121	102.48657	3,367	MH003724	MH070636	MH070889	MH071015	MH070762
*S. erubescens*	Luqu, Gansu	sn110814017, 353	34.59103	102.48699	3,345	MH003725	MH070637	MH070890	MH071016	MH070763
*S. erubescens*	Luqu, Gansu	sn110815020, 355	33.59203	101.48659	3,451	MH003726	MH070638	MH070891	MH071017	MH070764
*S. erubescens*	Xiahe, Gansu	Ikeda200713210, 371	35.20252	102.52181	3,342	MH003727	MH070639	MH070892	MH071018	MH070765
*S. globosa*	Aba, Sicuan	WYJ-2011-175, 109	33.63526	102.35556	3,470	MH003728	MH070640	MH070893	MH071019	MH070766
*S. globosa*	Baoxing, Sicuan	WYJ201607422, 168	30.49153	102.48188	3,992	MH003729	MH070641	MH070894	MH071020	MH070767
*S. globosa*	Kangding, Sicuan	WYJ201209151, 318	30.05441	101.96308	3,841	MH003730	MH070642	MH070895	MH071021	MH070768
*S. globosa*	Kangding, Sicuan	WYJ201209158, 329	30.05564	101.97304	3,864	MH003731	MH070643	MH070896	MH071022	MH070769
*S. globosa*	Kangding, Sicuan	WYJ201209157, 331	30.13242	101.56306	3,974	MH003732	MH070644	MH070897	MH071023	MH070770
*S. globosa*	–	–	–	–	–	EF420926	–	–	–	–
*S. globosa*	Xiangcheng, Sicuan	WYJ201209234, 337	28.93118	99.79842	3,764	MH003733	–	–	–	–
*S. globosa*	Xiangcheng, Sicuan	WYJ-2011-069, 80	28.53118	99.45658	3,835	MH003734	MH070645	MH070898	MH071024	MH070771
*S. globosa*	Xiangcheng, Sicuan	WYJ-2011-069, 81	28.53118	99.45658	3,835	MH003735	MH070646	MH070899	MH071025	MH070772
*S. involucrata*	Urumqi, Xinjiang	WYJ201607025a, 163	43.10847	86.84220	3,564	MH003736	MH070647	MH070900	MH071026	MH070773
*S. involucrata*	Urumqi, Xinjiang	WYJ201607025c, 165	43.10847	86.84220	3,564	MH003737	MH070648	MH070901	MH071027	MH070774
*S. involucrata*	Tekesi, Xinjiang	WYJ201308184, 24	43.09915	82.68382	3,678	MH003738	MH070649	MH070902	MH071028	MH070775
*S. involucrata*	Tekesi, Xinjiang	WYJ201308184, 26	43.09915	82.68382	3,678	MH003739	MH070650	MH070903	MH071029	MH070776
*S. involucrata*	Urumqi, Xinjiang	WYJ201308203, 372	43.11985	86.82125	3,768	MH003740	MH070651	MH070904	MH071030	MH070777
*S. involucrata*	Urumqi, Xinjiang	WYJ201308203, 374	43.11985	86.82125	3,768	MH003741	MH070652	MH070905	MH071031	MH070778
*S. involucrata*	Xinyuan, Xinjiang	WYJ201308188, 390	43.33469	84.01032	3,543	MH003742	MH070653	MH070906	MH071032	MH070779
*S. involucrata*	Urumqi, Xinjiang	WYJ201308203, 41	43.11985	86.82125	3,768	MH003743	MH070654	MH070907	MH071033	MH070780
*S. involucrata*	Xinyuan, Xinjiang	WYJ201308188, 47	43.33469	84.01032	3,543	MH003744	MH070655	MH070908	MH071034	MH070781
*S. involucrata*	Xinyuan, Xinjiang	WYJ201308188, 48	43.33469	84.01032	3,543	MH003745	MH070656	MH070909	MH071035	MH070782
*S. involucrata*	Dushanzi, Xinjiang	WYJ201308131, 61	43.77545	84.45615	2,684	MH003746	MH070657	MH070910	MH071036	MH070783
*S. involucrata*	Dushanzi, Xinjiang	WYJ201308131, 63	43.77545	84.45615	2,684	MH003747	MH070658	MH070911	MH071037	MH070784
*S. iodostegia*	Datong, Shanxi	WYJ201507117, 107	39.05578	113.65927	2,514	MH003748	MH070659	MH070912	MH071038	MH070785
*S. iodostegia*	Datong, Shanxi	WYJ201507117, 108	39.05578	113.65927	2,514	MH003749	MH070660	MH070913	MH071039	MH070786
*S. iodostegia*	Weixian, Hebei	WYJ201309004, 20	39.91413	114.96546	2,237	MH003750	MH070661	MH070914	MH071040	MH070787
*S. iodostegia*	Weixian, Hebei	WYJ201309004, 21	39.91413	114.96546	2,237	MH003751	MH070662	MH070915	MH071041	MH070788
*S. iodostegia*	Weixian, Hebei	WYJ201309004, 22	39.91413	114.96546	2,237	MH003752	MH070663	MH070916	MH071042	MH070789
*S. iodostegia*	Mentougou, Beijing	WYJ201507105, 27	40.03633	115.47206	2,048	MH003753	MH070664	MH070917	MH071043	MH070790
*S. iodostegia*	Mentougou, Beijing	WYJ201507105, 28	40.03633	115.47206	2,048	MH003754	MH070665	MH070918	MH071044	MH070791
*S. iodostegia*	Mentougou, Beijing	WYJ201507105, 29	40.03633	115.47206	2,048	MH003755	MH070666	MH070919	MH071045	MH070792
*S. luae*	Linzhi, Xizang	WYJ201607286a, 271	29.59022	94.59631	4,121	MH003756	–	–	–	–
*S. luae*	Linzhi, Xizang	WYJ201607286a, 272	29.59022	94.59631	4,121	MH003757	–	–	–	–
*S. luae*	Linzhi, Xizang	WYJ201607286b, 273	29.59022	94.59631	4,121	MH003758	MH070667	MH070920	MH071046	MH070793
*S. luae*	Linzhi, Xizang	WYJ201607286c, 283	29.59022	94.59631	4,121	MH003759	–	–	–	–
*S. luae*	Linzhi, Xizang	LJQ2620, 316	28.48051	93.36541	4,225	MH003760	MH070668	MH070921	MH071047	MH070794
*S. nigrescens*	Tianzhu, Gansu	LJQ1480, 314	36.41075	102.45620	1,900	MH003761	MH070669	MH070922	MH071048	MH070795
*S. nigrescens*	Sunan, Gansu	LJQ1517, 315	37.23345	102.32444	2,651	MH003762	MH070670	MH070923	MH071049	MH070796
*S. nigrescens*	Huangyuan, Qinghai	Liu1603, 320	36.20387	98.14870	3,700	MH003763	MH070671	MH070924	MH071050	MH070797
*S. nigrescens*	Huangzhong, Qinghai	WYJ200611, 347	36.50087	101.57164	3,641	MH003764	MH070672	MH070925	MH071051	MH070798
*S. nigrescens*	Menyuan, Qinghai	LJQ-QLS-2008-0065, 82	37.37502	101.62422	2,654	MH003765	MH070673	MH070926	MH071052	MH070799
*S. nigrescens*	Menyuan, Qinghai	LJQ-QLS-2008-0065, 83	37.37502	101.62422	2,654	MH003766	MH070674	MH070927	MH071053	MH070800
*S. nigrescens*	Menyuan, Qinghai	LJQ-QLS-2008-0065, 84	37.37502	101.62422	2,654	MH003767	MH070675	MH070928	MH071054	MH070801
*S. glandulosissima*	Chayu, Xizang	WYJ201607321, 257	29.32542	97.134728	3,949	MH003768	MH070676	MH070929	MH071055	MH070802
*S. glandulosissima*	Linzhi, Xizang	WYJ201607298, 264	29.627012	94.635744	4,433	MH003769	MH070677	MH070930	MH071056	MH070803
*S. glandulosissima*	Linzhi, Xizang	WYJ201607298, 379	29.627012	94.635744	4,433	MH003770	MH070678	MH070931	MH071057	MH070804
*S. glandulosissima*	Chayu, Xizang	WYJ201607321, 382	29.32542	97.134728	3,949	MH003771	MH070679	MH070932	MH071058	MH070805
*S. glandulosissima*	Chayu, Xizang	WYJ201607321, 383	29.32542	97.134728	3,949	MH003772	MH070680	MH070933	MH071059	MH070806
*S. orgaadayi*	Altay, Xinjiang	WYJ201308041, 11	47.21846	89.87999	3,541	MH003773	MH070681	MH070934	MH071060	MH070807
*S. orgaadayi*	Altay, Xinjiang	WYJ201308041, 12	47.21846	89.87999	3,541	MH003774	MH070682	MH070935	MH071061	MH070808
*S. orgaadayi*	Altay, Xinjiang	WYJ201308041, 360	47.21846	89.87999	3,541	MH003775	MH070683	MH070936	MH071062	MH070809
*S. phaeantha*	Xiaojing, Sicuan	WYJ201209126, 1	30.99918	102.3644	3,642	MH003776	MH070684	MH070937	MH071063	MH070810
*S. phaeantha*	Xiaojing, Sicuan	WYJ201209126, 2	30.99918	102.3644	3,642	MH003779	MH070687	MH070940	MH071066	MH070813
*S. phaeantha*	Qilian, Gansu	WYJ201607014, 195	38.60685	99.48221	4,096	MH003777	MH070685	MH070938	MH071064	MH070811
*S. phaeantha*	Qilian, Gansu	WYJ201607014, 196	38.60685	99.48221	4,096	MH003778	MH070686	MH070939	MH071065	MH070812
*S. phaeantha*	Maqin, Qinghai	LJQ1718, 317	34.47733	100.23956	3,210	MH003780	MH070688	MH070941	MH071067	MH070814
*S. phaeantha*	Xinghai, Qinghai	sn110718001, 349	35.58868	99.98818	2,654	MH003781	MH070689	MH070942	MH071068	MH070815
*S. phaeantha*	Xinghai, Qinghai	sn120811001, 351	34.32412	99.35641	2,641	MH003782	MH070690	MH070943	MH071069	MH070816
*S. phaeantha*	Xinghai, Qinghai	sn120801130, 354	35.38821	99.78935	2,684	MH003783	–	–	–	MH070817
*S. polycolea*	Linzhi, Xizang	WYJ201607292, 229	29.62701	94.63574	4,433	MH003784	MH070691	MH070944	MH071070	MH070818
*S. polycolea*	Linzhi, Xizang	WYJ201607292, 230	29.62701	94.63574	4,433	MH003785	MH070692	MH070945	MH071071	MH070819
*S. polycolea*	Linzhi, Xizang	WYJ201607292, 231	29.62701	94.63574	4,433	MH003786	MH070693	MH070946	MH071072	MH070820
*S. polycolea*	Langxian, Xizang	WYJ201607279, 269	28.883036	93.356181	4,472	MH003787	MH070694	MH070947	MH071073	MH070821
*S. polycolea*	Langxian, Xizang	WYJ201607279, 270	28.883036	93.356181	4,472	MH003788	MH070695	MH070948	MH071074	MH070822
*S. polycolea*	Linzhi, Xizang	Liu07257, 334	29.62201	94.63554	4,231	MH003789	MH070696	MH070949	MH071075	MH070823
*S. pubifolia*	Jiacha, Xizang	WYJ201607272a, 206	29.03175	92.35724	4,796	MH003790	MH070697	MH070950	MH071076	MH070824
*S. pubifolia*	Jiacha, Xizang	WYJ201607272b, 207	29.03175	92.35724	4,796	MH003791	MH070698	MH070951	MH071077	MH070825
*S. pubifolia*	Jiacha, Xizang	WYJ201607272c, 208	29.03175	92.35724	4,796	MH003792	MH070699	MH070952	MH071078	MH070826
*S. pubifolia*	Jiacha, Xizang	WYJ-2011-057, 94	29.02165	92.35714	4,786	MH003793	MH070700	MH070953	MH071079	MH070827
*S. sikkimensis*	Cuona, Xizang	WYJ201607242, 156	27.92057	91.84863	3,970	MH003794	MH070701	MH070954	MH071080	MH070828
*S. sikkimensis*	Yadong, Xizang	WYJ201607150e, 186	27.48592	88.90708	4,102	MH003795	MH070702	MH070955	MH071081	MH070829
*S. sikkimensis*	Yadong, Xizang	WYJ201607150c, 187	27.48592	88.90708	4,102	MH003796	MH070703	MH070956	MH071082	MH070830
*S. sikkimensis*	Yadong, Xizang	WYJ201607150f, 385	27.48592	88.90708	4,102	MH003797	MH070704	MH070957	MH071083	MH070831
*S. sikkimensis*	Yadong, Xizang	WYJ201607150 h, 386	27.48592	88.90708	4,102	MH003798	MH070705	MH070958	MH071084	MH070832
*S. sikkimensis*	Cuona, Xizang	WYJ201607242, 388	27.92057	91.84863	3,970	MH003799	MH070706	MH070959	MH071085	MH070833
*S. sikkimensis*	Cuona, Xizang	WYJ201607242, 389	27.92057	91.84863	3,970	MH003800	MH070707	MH070960	MH071086	MH070834
*S. tangutica*	Qilian, Gansu	WYJ201607013, 226	38.60685	99.48221	4,096	MH003801	MH070708	MH070961	MH071087	MH070835
*S. tangutica*	Qilian, Gansu	WYJ201607013, 228	38.60685	99.48221	4,096	MH003802	MH070709	MH070962	MH071088	MH070836
*S. tangutica*	Zhiduo, Qinghai	WYJ201207279, 328	33.85203	95.61335	3,948	MH003803	MH070710	MH070963	MH071089	MH070837
*S. tangutica*	Kangding, Sicuan	sn120801019, 332	30.05093	101.96437	3,987	MH003804	MH070711	MH070964	MH071090	MH070838
*S. tangutica*	Kangding, Sicuan	sn120801019, 335	30.05093	101.96437	3,987	MH003805	MH070712	MH070965	MH071091	MH070839
*S. tangutica*	Zhiduo, Qinghai	WYJ201207279, 340	33.85203	95.61335	3,948	MH003806	MH070713	MH070966	MH071092	MH070840
*S. uniflora*	Cuona, Xizang	WYJ201607254, 142	27.765831	91.90194	4,138	MH003807	MH070714	MH070967	MH071093	MH070841
*S. uniflora*	Cuona, Xizang	WYJ201607254, 143	27.765831	91.90194	4,138	MH003808	MH070715	MH070968	MH071094	MH070842
*S. uniflora*	Cuona, Xizang	WYJ201607254, 144	27.765831	91.90194	4,138	MH003809	MH070716	MH070969	MH071095	MH070843
*S. uniflora*	Yadong, Xizang	WYJ201607151c, 145	27.48592	88.90708	4,102	MH003810	MH070717	MH070970	MH071096	MH070844
*S. uniflora*	Yadong, Xizang	WYJ201607151a, 146	27.48592	88.90708	4,102	MH003811	MH070718	MH070971	MH071097	MH070845
*S. uniflora*	Yadong, Xizang	WYJ201607151b, 147	27.48592	88.90708	4,102	MH003812	–	–	–	–
*S. uniflora*	Cuona, Xizang	WYJ201607243, 197	27.92057	91.84863	3,970	MH003813	MH070719	MH070972	MH071098	MH070846
*S. veitchiana*	Xinglong, Hebei	WYJ201507098, 302	40.59808	117.47655	2,032	MH003814	MH070720	MH070973	MH071099	MH070847
*S. veitchiana*	Xinglong, Hebei	WYJ201507098, 303	40.59808	117.47655	2,032	MH003815	MH070721	MH070974	MH071100	MH070848
*S. veitchiana*	Nuanchuan, Henan	WYJ201507135, 52	33.67057	111.79417	1,651	MH003816	MH070722	MH070975	MH071101	MH070849
*S. veitchiana*	Nuanchuan, Henan	WYJ201507135, 53	33.67057	111.79417	1,651	MH003817	MH070723	MH070976	MH071102	MH070850
*S. veitchiana*	Nuanchuan, Henan	WYJ201507135, 54	33.67057	111.79417	1,651	MH003818	MH070724	MH070977	MH071103	MH070851
*S. veitchiana*	Nuanchuan, Henan	WYJ201507135, 55	33.67057	111.79417	1,651	MH003819	MH070725	MH070978	MH071104	MH070852
*S. veitchiana*	Shenlongjia, Hubei	WYJ201507160, 57	31.43997	110.307149	3,098	MH003820	MH070726	MH070979	MH071105	MH070853
*S. veitchiana*	Shenlongjia, Hubei	WYJ201507160, 58	31.43997	110.307149	3,098	MH003821	MH070727	MH070980	MH071106	MH070854
*S. veitchiana*	Shenlongjia, Hubei	WYJ201507160, 59	31.43997	110.307149	3,098	MH003822	MH070728	MH070981	MH071107	MH070855
*S. veitchiana*	Wuxi, Chongqing	WYJ201507184, 64	31.43791	109.15498	1,795	MH003823	MH070729	MH070982	MH071108	MH070856
*S. veitchiana*	Wuxi, Chongqing	WYJ201507184, 65	31.43791	109.15498	1,795	MH003824	MH070730	MH070983	MH071109	MH070857
*S. veitchiana*	Wuxi, Chongqing	WYJ201507184, 66	31.43791	109.15498	1,795	MH003825	MH070731	MH070984	MH071110	MH070858
*S. veitchiana*	Wuxi, Chongqing	WYJ201507184, 67	31.43791	109.15498	1,795	MH003826	MH070732	MH070985	MH071111	MH070859
*S. velutina*	Xiaojin, Sichuan	WYJ201209124, 339	30.99441	102.82915	4,000	MH003827	MH070733	MH070986	MH071112	MH070860
*S. velutina*	Xiaojin, Sichuan	WYJ201209124, 342	30.99441	102.82915	4,000	MH003828	MH070734	MH070987	MH071113	MH070861
*S. velutina*	Xiaojin, Sichuan	WYJ201209124, 76	30.99441	102.82915	4,000	MH003829	MH070735	MH070988	MH071114	MH070862
*S. velutina*	Xiaojin, Sichuan	WYJ201209124, 77	30.99441	102.82915	4,000	MH003830	MH070736	MH070989	MH071115	MH070863
*S. velutina*	Xiaojin, Sichuan	WYJ201209124, 78	30.99441	102.82915	4,000	MH003831	MH070737	MH070990	MH071116	MH070864
*S. wettsteiniana*	Mianning, Sichuan	WYJ201607408a, 176	29.00106	102.14985	3,381	MH003832	MH070738	MH070991	MH071117	MH070865
*S. wettsteiniana*	Mianning, Sichuan	WYJ201607408b, 177	29.00106	102.14985	3,381	MH003833	MH070739	MH070992	MH071118	MH070866
*S. wettsteiniana*	Mianning, Sichuan	WYJ201607402, 178	29.00106	102.14985	3,381	MH003834	MH070740	MH070993	MH071119	MH070867
*S. wettsteiniana*	Mianning, Sichuan	WYJ201607402, 284	29.00106	102.14985	3,381	MH003835	MH070741	MH070994	MH071120	MH070868
*Jurinea multiflora*	Tuoli, Xinjiang	WYJ201308102, 377	45.73564	83.14712	1,753	MH003704	MH070616	MH070869	MH070995	MH070742

### DNA extraction, PCR amplification, and sequencing

Genomic DNA was extracted from dried leaves in silica gel using the CTAB method (Doyle, 1987). Five regions (*rbc*L, *mat*K, *trn*H-*psb*A, *trn*K, and ITS) (Berends, Jones & Mullet, 1990; Ford et al., 2009; Olmstead et al., 1992; Sang, Crawford & Stuessy, 1997; White et al., 1990), were amplified and sequenced using the primers listed in Table 3. A PCR reaction mixture comprising 25 µL was prepared and amplified according to the procedure described by Wang et al. (2009). The PCR products were sent to the Beijing Genomics Institute for commercial sequencing. Sequences were aligned using CLUSTALX v.2.1 (Thompson et al., 1997) with the default settings and adjusted manually with Bioedit v.7.0.5 (Hall, 1999). All of the sequences were registered in GenBank (Table 2).

**Table 3:** List of the primers used in this study.

Primer	Fragment	Sequence(5′–3′)	Reference
ITS4	ITS	TCCTCCGCTTATTGATATGC	White et al. (1990)
ITS1	ITS	AGAAGTCGTAACAAGGTTTCCGTAGG	White et al. (1990)
*trn*K(UUU)	*trn*K	TTAAAAGCCGAGTACTCTACC	Berends, Jones & Mullet (1990)
*rps*16	*trn*K	AAAGTGGGTTTTTATGATCC	Berends, Jones & Mullet (1990)
*psb*A	*psb*A	GTTATGCATGAACGTAATGCTC	Sang, Crawford & Stuessy (1997)
*trn*H	*psb*A	CGCGCATGGTGGATTCACAATCC	Sang, Crawford & Stuessy (1997)
*mat*K-xf	*mat*K	TAATTTACGATCAATTCATTC	Ford et al. (2009)
*mat*K-5r	*mat*K	GTTCTAGCACAAGAAAGTCG	Ford et al. (2009)
*rbc*L1	*rbc*L	ATGTCACCACAAACAGAGACTAAAGC	Olmstead et al. (1992)
*rbc*L911	*rbc*L	TTTCTTCGCATGTACCCGC	Olmstead et al. (1992)

### Data analysis

We constructed 31 datasets for ITS, *psb*A-*trn* H, *mat*K, and *trn*K, either individually or in different combinations. For the combination of ITS and each chloroplast loci, incongruence length difference (ILD) was preferred to test the incongruence (Farris et al., 1995) using PAUP version 4b10 (Swofford, 2003). For each dataset, the inter- and intraspecific genetic divergences were calculated as described by Meyer & Paulay (2005) and used to determine whether a barcoding gap was present. For each dataset, best close match (BCM) and two tree-based methods comprising neighbor-joining (NJ) and Bayesian inference (BI) were employed to analyze the five single markers and their different combinations. BCM analysis was conducted using the SPIDER package in R (Brown et al., 2012). NJ trees were constructed using PAUP with the Kimura two-parameter model (Swofford, 2003). Support for nodes was assessed based on 100,000 bootstrap replicates. BI analysis was implemented using MrBayes on XSEDE (v3.2.6) (Ronquist et al., 2012) and the optimal models for each marker were determined according to Akaike’s information criterion with jModelTest2 in XSEDE (v2.1.6) (Darriba et al., 2012). Species were considered to be identified successfully if individual samples of a species clustered in species-specific monophyletic clades.

## Results

**Figure 2:**
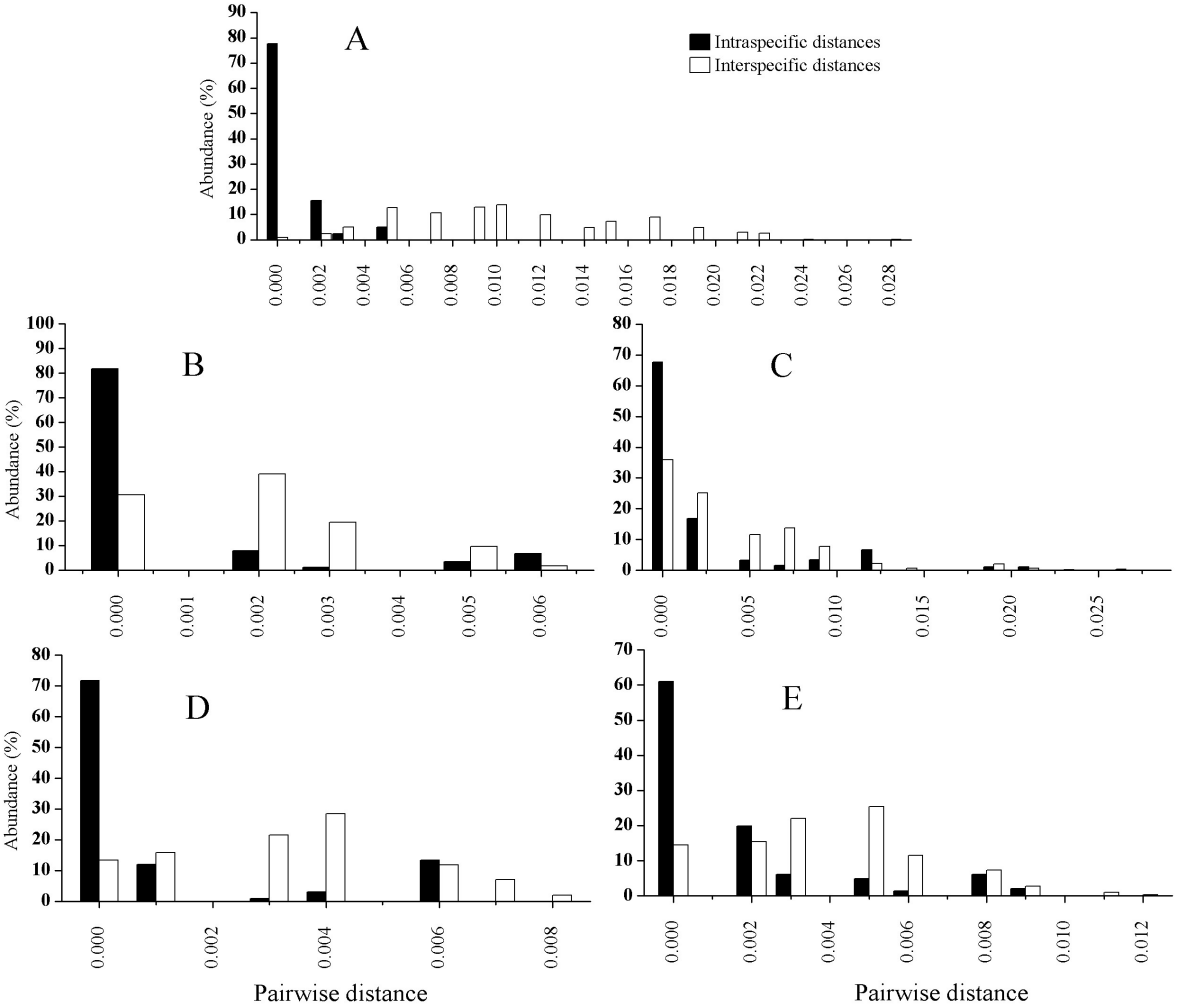
Relative distributions of intraspecific and interspecific distances calculated with ITS (A), *rbc*L (B), *trn*H-*psb*A (C), *mat*K (D), and *trn*K (E).

The PCR amplification ranged from about 73% (*trn*K) to 93% (ITS), while sequencing success rates from about 95% for the three chloroplast loci to 100% for the ITS, as shown in Table 4. The length after alignment, the variable sites, the interspecific or intraspecific genetic distance for each locus as well as the *p* values of ILD test between ITS and each chloroplast locus are also listed in Table 4. The mean intraspecific genetic distances for each species based on ITS and the four cp markers combined are listed in Table 5, and those for the mean interspecific genetic distances are shown in Table 6. The distributions of the intraspecific and interspecific distances for each species based on the five separate markers are shown in Fig. 2. In general, the mean interspecific distances were higher than the intraspecific distances for the five markers. However, the ranges of the intra- and interspecific distances overlapped for all the barcodes tested in this study.

**Table 4:** List of statistics information of five DNA barcodes and the result of incongruence length difference (ILD) analysis between ITS and each chloroplast locus.

DNA region	ITS	*trn*H-*psb*A	*mat*K	*rbc*L	*trn*K
PCR success (%)	92.7	77	89.6	91.6	72.9
Sequencing success (%)	100	96.18	95.42	95.42	95.42
Aligned sequence length (bp)	656	444	711	634	656
No. indel (length in bp)	3 (1)	5 (1–3)	0	0	4 (1)
No. variated sites	111	22	18	8	28
No. sampled species (individual)	19 (131)	19 (131)	19 (131)	19 (131)	19 (131)
Interspecific distance mean (range) (%)	0.011 (0-0.028)	0.004(0–0.028)	0.003(0–0.008)	0.002(0–0.006)	0.004(0–0.012)
Intraspecific distance mean (range) (%)	0.001(0–0.005)	0.002(0–0.021)	0.001(0–0.006)	0.001(0–0.006)	0.001(0–0.009)
*p* values of ILD test between ITS	–	0.02	0.001	0.12	0.001

**Table 5:** Mean intraspecies distance (%) of ITS and the combined sequences of four chloroplast loci for each species.

Species	ITS	Chloroplast
*S. bogedaensis*	0.0	0.02
*S. bracteata*	0.0	0.00
*S. erubescens*	0.0	0.00
*S. glandulosissima*	0.1	0.07
*S. globosa*	0.2	0.04
*S. involucrata*	0.2	0.06
*S. iodostegia*	0.0	0.05
*S. luae*	0.0	0.29
*S. nigrescens*	0.0	0.00
*S. orgaadayi*	0.0	0.00
*S. phaeantha*	0.4	0.04
*S. polycolea*	0.0	0.07
*S. pubifolia*	0.0	0.00
*S. sikkimensis*	0.2	0.06
*S. tangutica*	0.1	0.46
*S. uniflora*	0.1	0.15
*S. veitchiana*	0.1	0.39
*S. velutina*	0.0	0.21
*S. wettsteiniana*	0.0	0.00

**Table 6:** The pairwise distances (%) of ITS (lower left) and the combined chloroplast loci (upper right) from 19 species of *Saussurea*. (1) *S. bogedaensis*, (2) *S. bracteata*, (3) *S. erubescens*, (4) *S. globosa*, (5) *S. involucrate*, (6) *S. iodostegia*, (7) *S. luae*, (8) *S. nigrescens*, (9) *S. glandulosissima*, (10) *S. orgaadayi*, (11) *S. phaeantha*, (12) *S. polycolea*, (13) *S. pubifolia*, (14) *S. sikkimensis*, (15) *S. tangutica*, (16) *S. uniflora*, (17) *S. veitchiana*, (18) *S. velutina*, (19) *S. wettsteiniana*.

CP ITS	1	2	3	4	5	6	7	8	9	10	11	12	13	14	15	16	17	18	19
1		0.30	0.26	0.28	0.22	0.62	0.32	0.34	0.28	0.22	0.28	0.34	0.30	0.41	0.46	0.34	0.55	0.34	0.26
2	1.92		0.04	0.06	0.17	0.57	0.19	0.29	0.22	0.16	0.06	0.12	0.00	0.35	0.35	0.23	0.50	0.16	0.21
3	1.52	2.77		0.02	0.13	0.53	0.14	0.25	0.18	0.12	0.02	0.08	0.04	0.31	0.31	0.19	0.46	0.12	0.16
4	1.53	2.88	0.61		0.15	0.55	0.17	0.27	0.20	0.15	0.05	0.10	0.06	0.34	0.33	0.22	0.48	0.15	0.19
5	0.93	2.58	2.14	2.14		0.48	0.19	0.21	0.14	0.09	0.15	0.20	0.17	0.27	0.33	0.21	0.42	0.20	0.13
6	1.96	3.33	1.85	1.60	2.47		0.59	0.53	0.54	0.49	0.55	0.60	0.57	0.51	0.71	0.55	0.37	0.57	0.53
7	1.07	0.72	1.90	1.78	1.72	2.31		0.31	0.18	0.19	0.17	0.21	0.19	0.37	0.39	0.25	0.52	0.23	0.23
8	1.83	3.19	1.72	1.47	2.34	0.34	2.12		0.26	0.21	0.27	0.32	0.29	0.31	0.45	0.22	0.32	0.19	0.25
9	1.35	2.69	1.56	1.31	1.92	1.74	1.69	1.60		0.14	0.20	0.24	0.22	0.33	0.34	0.22	0.47	0.26	0.18
10	1.41	3.08	2.30	2.35	2.02	2.28	2.21	2.17	2.16		0.15	0.20	0.16	0.27	0.32	0.21	0.42	0.20	0.12
11	1.53	2.84	1.60	1.45	2.14	1.92	1.84	1.78	1.31	2.34		0.10	0.06	0.34	0.33	0.22	0.48	0.15	0.19
12	1.09	2.42	1.36	1.06	1.69	1.48	1.43	1.35	0.87	1.89	0.89		0.12	0.37	0.37	0.26	0.53	0.20	0.24
13	1.61	1.32	2.22	2.23	2.26	3.00	0.23	2.84	2.37	2.76	2.51	2.10		0.35	0.35	0.23	0.50	0.16	0.21
14	1.11	2.44	1.34	1.08	1.71	1.49	1.38	1.36	0.71	1.91	1.07	0.64	2.12		0.51	0.34	0.48	0.35	0.31
15	1.63	2.98	1.58	1.59	1.47	2.57	2.01	2.42	2.06	2.67	2.20	1.78	2.32	1.81		0.42	0.65	0.40	0.35
16	1.00	2.33	1.27	0.97	1.44	1.38	1.34	1.26	0.78	1.80	0.96	0.53	2.01	0.55	1.70		0.46	0.24	0.25
17	2.10	3.48	2.06	1.74	2.62	1.52	2.36	1.30	1.72	2.93	2.02	1.62	2.81	1.64	2.50	1.53		0.45	0.46
18	2.21	2.91	2.49	2.50	2.50	2.94	2.04	2.80	2.31	3.04	2.50	2.05	2.59	2.07	2.66	1.96	3.09		0.24
19	1.73	3.05	1.88	1.70	2.35	1.80	1.85	1.69	1.19	2.39	1.65	1.25	2.77	1.09	2.45	1.16	2.27	2.71	

The discriminatory powers of all the loci both individually and in different combinations based on the three methods are listed in Table 7 (Figs. S1–S59). In general, BCM achieved higher success rates, followed by NJ and BI, but there were a few exceptions. Among the results obtained with a single barcode, ITS (84.2–93.2%) had the highest species discriminatory power, followed by *trn*K (15.8–36%), *mat*K (10.5–16.8%), and *trn*H-*psb*A (5.2–27%). Among the combinations of two barcodes, ITS + *rbc*L had the highest discriminatory success (89.5–100%), whereas that of *mat*K and *rbc*L, which was suggested as the core barcode by CBOL (CBOL Plant Working Group, 2009), was only 10.5–25.6%. The three-region combination of ITS + *rbc*L + *trn*H-*psb*A recovered the highest number of monophyletic species (18) in the NJ tree (94.7%). Only five species were successfully discriminated (26.3%) by either the NJ or BI trees using the combination of all four cp markers, i.e., *mat*K + *rbc*L + *trn*H-*psb*A + *trn*K.

**Table 7:** Species resolution using the Best Close Match method and the tree-based method with five barcodes and their combinations.

Sequences	Number	Best close match (%)					BI (%)	NJ (%)
		Correct	Ambiguous	Incorrect	No match	Threshold		
ITS	132	93.2	6.8	0.0	0.0	0.45	84.2	84.2
*trn*K	125	36.0	61.6	2.4	0.0	0.91	15.8	15.8
*mat*K	125	16.8	83.2	0.0	0.0	0.56	10.5	10.5
*psb*A	126	27.0	71.4	0.8	0.8	1.12	5.2	5.2
*rbc*L	125	12.0	88.0	0.0	0.0	0.63	0.0	0.0
ITS+*trn*K	125	98.4	0.0	1.6	0.0	0.53	79.0	84.2
ITS+matk	125	96.0	3.2	0.8	0.0	0.36	79.0	84.2
ITS+*psb*A	126	96.0	4.0	0.0	0.0	0.54	84.2	89.5
ITS+*rbc*L	125	100.0	0.0	0.0	0.0	0.38	89.5	89.5
trnK+*mat*K	125	52.0	45.6	2.4	0.0	0.72	26.3	26.3
*trn*K+*psb*A	125	52.0	44.8	3.2	0.0	0.99	21.1	21.1
*trn*K+*rbc*L	125	37.6	60.8	1.6	0.0	0.77	15.8	15.8
*mat*K+*psb*A	125	49.6	48.8	1.6	0.0	0.77	21.1	15.8
*mat*K+*rbc*L	125	25.6	74.4	0.0	0.0	0.59	10.5	10.5
*psb*A+*rbc*L	125	30.4	68.8	0.8	0.0	0.83	10.5	5.2
ITS+*mat*K+*psb*A	125	96.0	3.2	0.8	0.0	0.54	68.4	89.5
ITS+*trn*K+*mat*K	125	98.4	0.0	1.6	0.0	0.54	73.7	89.5
ITS+*trn*K+*rbc*L	125	98.4	0.0	1.6	0.0	0.51	84.2	89.5
ITS+*mat*K+*rbc*L	125	99.2	0.0	0.8	0.0	0.39	79.0	89.5
ITS+*rbc*L+*psb*A	125	100.0	0.0	0.0	0.0	0.57	79.0	94.7
ITS+*trn*K+*psb*A	125	98.4	0.0	1.6	0.0	0.68	79.0	89.5
*trn*K+*mat*K+*rbc*L	125	52.0	45.6	2.4	0.0	0.69	26.3	26.3
*trn*K+*mat*K+*psb*A	125	63.2	35.2	1.6	0.0	0.82	26.3	26.3
*mat*K+*psb*A+*rbc*L	125	49.6	49.6	0.8	0.0	0.72	21.1	21.1
*rbc*L+*trn*K+*psb*A	125	55.2	41.6	3.2	0.0	0.86	15.8	21.1
ITS+*mat*K+*psb*A+*rbc*L	125	99.2	0.0	0.8	0.0	0.57	68.4	84.2
ITS+*mat*K+*psb*A+*trn*K	125	98.4	0.0	1.6	0.0	0.64	73.7	84.2
ITS+*mat*K+*rbc*L+*trn*K	125	98.4	0.0	1.6	0.0	0.52	73.7	84.2
ITS+*rbc*L+*trn*K+*psb*A	125	98.4	0.0	1.6	0.0	0.66	79.0	84.2
*trn*K+*mat*K+*psb*A+*rbc*L	125	63.2	35.2	1.6	0.0	0.77	26.3	26.3
ITS+*trn*K+*mat*K+*psb*A+*rbc*L	125	98.4	0.0	1.6	0.0	0.64	79.0	84.2

## Discussion

### Proposed DNA barcodes for *S.* subg. *Amphilaena*

**Figure 3:**
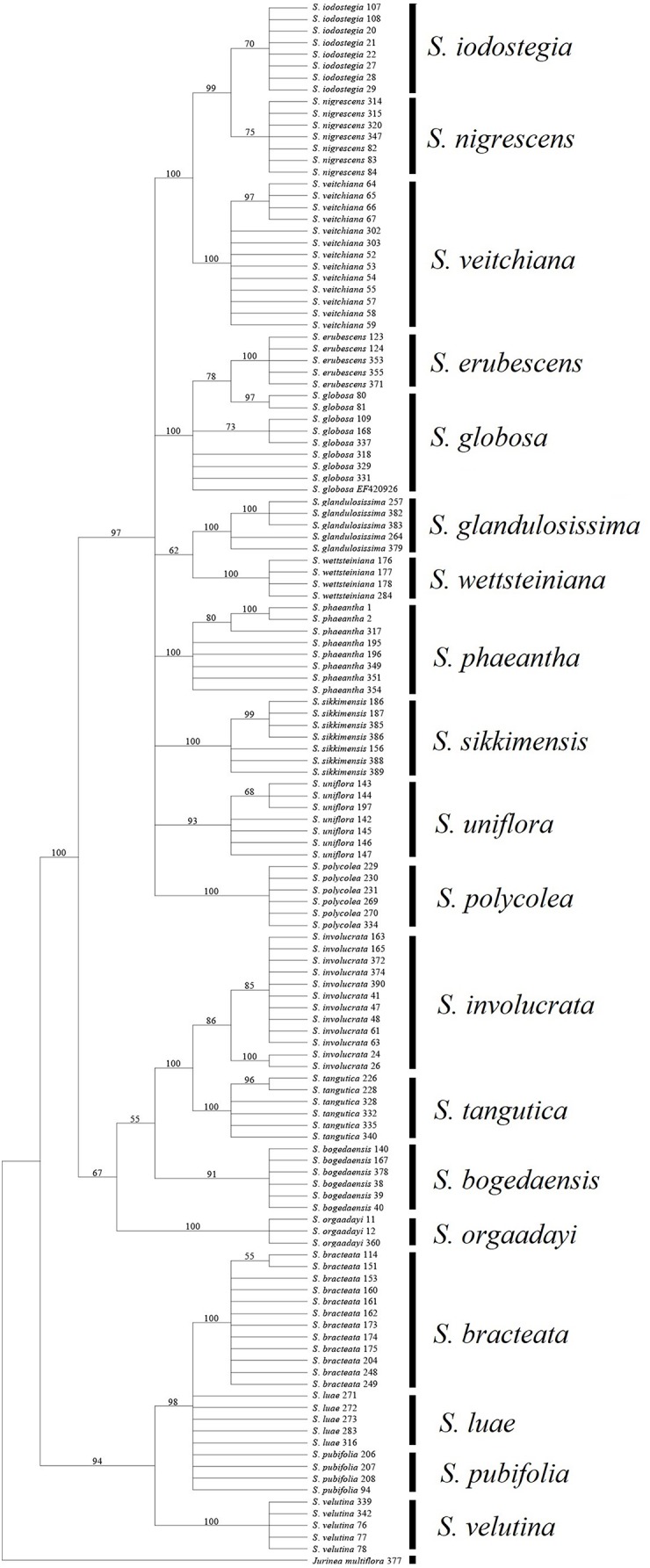
Phylogenetic tree based on Bayesian analysis of ITS.

Among the fragments tested in the present study, ITS obtained a much higher success rate compared with the other loci. In addition, all of the combinations without ITS yielded much lower success rates, regardless of the method used (Table 7). Moreover, the rate of successful PCR (92.7%) was more or less higher for ITS than the other fragments (72.9–91.6%). It has also been reported that this fragment is highly efficient in other Asteraceae genera (Gao et al., 2010; Gong et al., 2016). However, an intrinsic problem with this fragment is that an individual may have undergone recent hybridization, thereby resulting in multiple mosaic sites (Li et al., 2011). In *S.* subg. *Amphilaena*, two species failed to form monophyletic clades in the BI and NJ trees, which could be attributed to the presence of multiple mosaic sites (Fig. 3). However, ITS performed better than the other fragments in *S.* subg. *Amphilaena*, and thus we propose that this fragment should be the first or best choice when selecting only one of the current candidates.

**Figure 4:**
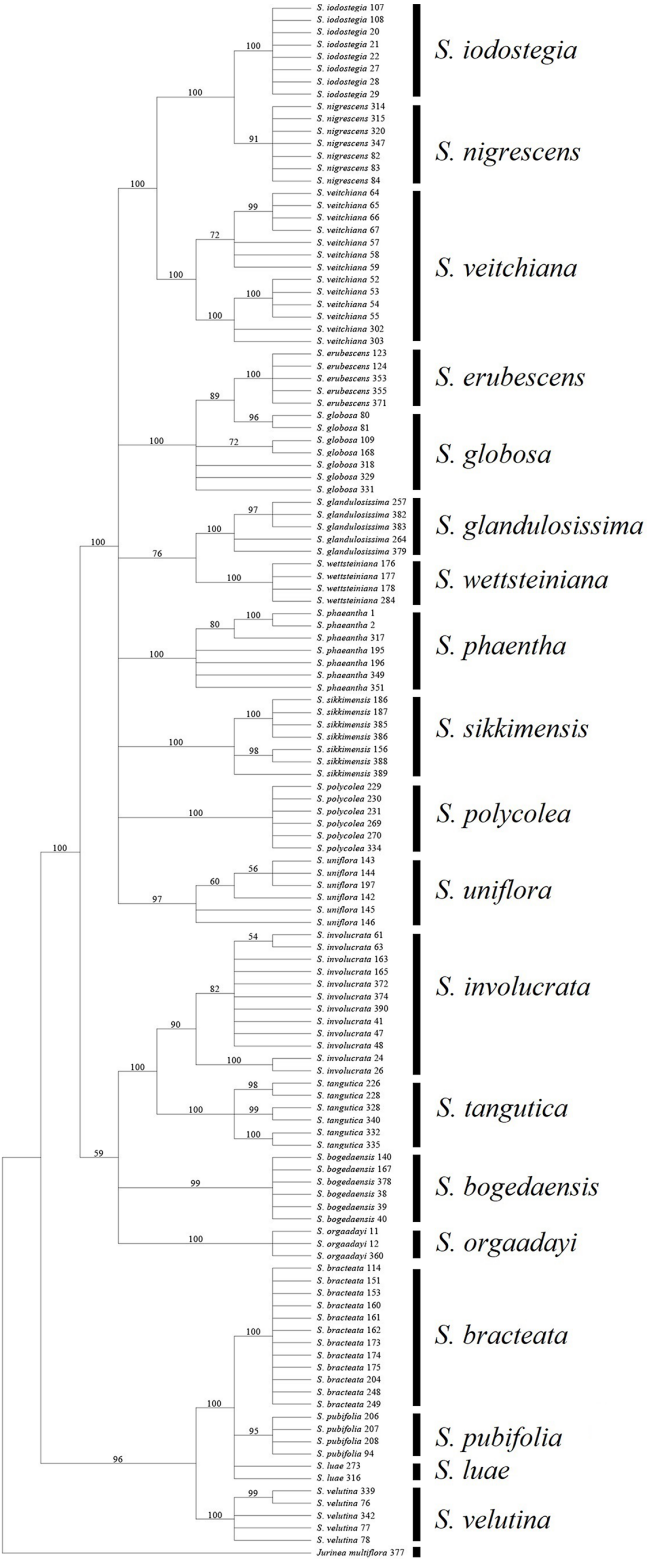
Phylogenetic tree based on Bayesian analysis of ITS + *rbc*L.

We found that it was difficult to identify the best second choice after ITS. *Trn*K performed much better than *rbc*L in terms of its efficiency when used individually, but its combination with ITS obtained contradictory results, i.e., ITS + *trn*K was inferior to ITS + *rbc*L in terms of efficiency. This contradictory result was unexpected and it is not common in other taxa (Cao et al., 2010; Müller & Borsch, 2005). We attributed this result to higher degree of congruence of the concatenated sequences of *rbc*L and ITS (*P* = 0.12 for ILD test), in compare to *trn*K and ITS (*P* = 0.001). But it might derive from some other mechanisms, such as the higher rate of mutation for *trn*K that could have caused differentiation within species, but not high enough to form distinct genetic differentiation among species, and thus a failure to cluster as a monophyletic group in line with species (Naciri, Caetano & Salamin, 2012; Petit & Excoffier, 2009). Therefore, we suggest that using *trn*K alone is problematic and instead we propose to use *rbc*L as complementary to ITS because this combination could identify all 19 of the sampled species based BCM, and 17 by NJ or BI (89%) (Table 7) (Fig. 4).

The two loci comprising *trn*H -*psb*A and *mat*K were affected by the same problem as *trn*K, with higher mutation rates and barcode efficiencies compared with *rbc*L when used individually, but lower efficiency when combined with ITS. Thus, their combination with ITS + *rbc*L failed to significantly increase the success rate and lower results were even obtained in some cases (Table 7). However, among the combinations without ITS, the combination with higher mutation rates was more efficient than those with lower mutation rates, e.g., *trn*K *+ trn*H*-psb*A was better than *mat*K *+ rbc*L, which was proposed previously as the core DNA barcode for plants (Hollingsworth et al., 2009). Therefore, if ITS is subjected to hybridization, we propose that the priority order should be the following: *trn*K > *trn*H*-psb*A >  *mat*K > *rbc*L*.* Moreover, the combination with more loci performed better than that with less loci. However, even the combination of all four loci was not sufficient to discriminate each species and new fragments should be considered.

### Insights into taxonomic problems based on DNA barcodes

**Figure 5:**
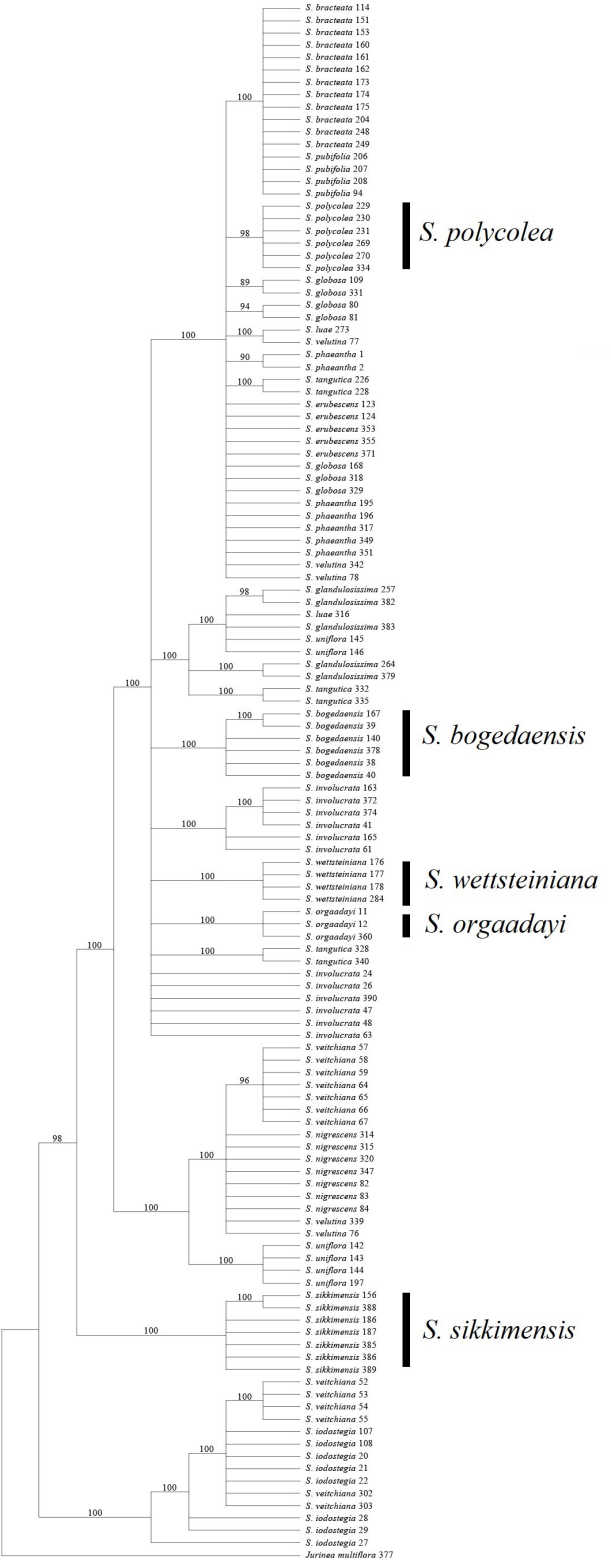
Phylogenetic tree based on Bayesian analysis of *trn*K +*mat*K +*psb*A +*rbc*L.

Most of the analyses failed to identify the species within two groups, i.e., *S. luae* vs. *S. publifolia* and *S. globosa* vs. *S. erubescens* (Figs. 3–5; Table 7). We found that these failures might have been attributable to taxonomic problems. For the first group, we found that *S. luae* was rather heterogeneous in terms of the ITS sequences. Some cp sequences were slightly differentiated compared with *S. velutina*, but the others were closer to those in *S. glandulosissima* or *S. uniflora* (Fig. 5). By contrast, the ITS sequences lacked variance and after excluding the mosaic sites, they were closely related in *S. pubifolia* or *S. bracteata* (Fig. 3). These nuclear-cytoplasmic inconsistencies suggest that hybridization may have occurred among these species.

The second group comprising *S. globosa* and *S. erubescens* was often confused in previous studies because the latter resembles a smaller form of *S. globosa*, which has various forms across its distribution (Raab-Straube, 2017). In agreement with the morphology, the genetic distance between the cp sequences within *S. erubescens* was zero whereas that within *S. globosa* was 0.04% (Table 5), which is even larger than that between *S. erubescens* and *S. globosa* (Table 6). The ITS sequences had a very similar pattern and the rich mosaic sites in both species also indicated differentiation accompanying substantial gene flow (Naciri, Caetano & Salamin, 2012). Both the BI and NJ methods found that *S. globosa* formed a clade within which *S. erubescens* nested as a monophyletic clade (Fig. 3). Based on these results, we propose that *S. globosa* might be a species with a series of differentiated populations where *S. erubescens* represents one of the most obvious. The current delimitation might need revision on the basis of extensive morphological as well as genetic diversity across the distribution range of both species.

### Identification of the medicinal species and the potential substitutes

All of the known medically important species could be identified using our proposed DNA barcodes, i.e., ITS + *rbc*L or ITS alone (Table 7; Figs. 3–4). Moreover, some species such as *S. bogedaensis*, *S. glandulosissima*, *S. polycolea*, *S. wettsteiniana*, and *S. orgaadayi* could be identified with the cp DNA barcodes (Fig. 5). This high rate of success was unexpected because some species such as the two species in the *S. obvallata* complex (*S. glandulosissima* and *S. sikkimensis*) have been morphologically confused for many years and they were only separated very recently (Raab-Straube, 2017). Their distinction is indicative of difference in bioactive components. Therefore, our results caution against their indiscriminating usage in medicine.

Barcode sequences can also help to identify substitutes for medically useful species because closely related species might possibly share the same or similar secondary metabolites and bioactivities (Zhou et al., 2014). Thus, we propose that nine of the 15 medically useful species might be substituted by their close relatives according to the molecular phylogenetic context. Six of these species, which formed three groups, are also morphologically similar, i.e., *S. involucrata* and *S. orgaadayi* or *S. bogedaensis*, *S. globosa* and *S. erubescens*, and *S. wettsteiniana* and *S. glandulosissima* (Fig. 3) (Raab-Straube, 2017). Among the remaining three species, *S. bracteata* appears to be closely related to *S. pubifolia* whereas *S. iodostegia* and *S. nigrescens* are closely related to each other according to phylogenetic tree (Fig. 3). These affinities were not expected according to their morphology, but they are possibly due to convergent evolution or radiation in *Saussurea* (Wang et al., 2009). Secondary metabolomes or bioactivities are wanted to confirm their similarity.

## Conclusion

Based on the sequence statistics, inter- and intraspecific distances, SPIDER, and phylogenetic analyses, it is concluded that internal transcribed spacer (ITS) + *rbc*L or ITS + *rbc*L + *psb*A-*trn*H could distinguish all of the species, while the ITS alone could identify all of the 15 medical plants. However, the species identification rates based on plastid barcodes were low, i.e., 0% to 36% when analyzed individually, and 63% when all four loci were combined. Thus, we recommend using ITS + *rbc*L as the DNA barcode for *S.* subg. *Amphilaena* or the ITS alone for medical plants.

##  Supplemental Information

10.7717/peerj.6357/supp-1 Supplemental Information 1 BI and NJ trees of different combinations of DNA regions areas

10.7717/peerj.6357/supp-2 Supplemental Information 2 psbA sequences in FASTA format

10.7717/peerj.6357/supp-3 Supplemental Information 3 matK sequences in FASTA format

10.7717/peerj.6357/supp-4 Supplemental Information 4 rbcL sequences in FASTA format

10.7717/peerj.6357/supp-5 Supplemental Information 5 trnK sequences in FASTA format

10.7717/peerj.6357/supp-6 Supplemental Information 6 ITS sequences in FASTA format

## Additional Information and Declarations

### Data Availability

The following information was supplied regarding data availability: All of the sequences used in this article are registered in GenBank: accession numbers MH003704 to MH003835 for ITS and MH070616 to MH071120 for chloroplast regions.
