# Supplementary material for: Identification of species and materia medica within Saussurea subg. Amphilaena based on DNA barcodes
Source: PeerJ. 2019 Feb 1;7:e6357. doi: 10.7717/peerj.6357 (PMC6361007; doi:10.7717/peerj.6357)
Supplement: Supplemental Information 1 [file peerj-07-6357-s001.zip › Supporting Information/Figure legends.pdf]

Fig. S1. BI tree based on *mat*K.
Fig. S2. BI tree based on *psb*A.
Fig. S3. BI tree based on *rbc*L.
Fig. S4. BI tree based on *trn*K.
Fig. S5. BI tree based on ITS+*mat*K.
Fig. S6. BI tree based on ITS+*psb*A.
Fig. S7. BI tree based on ITS+*trn*K.
Fig. S8. BI tree based on *mat*K+*psb*A.
Fig. S9. BI tree based on *mat*K+*rbc*L.
Fig. S10. BI tree based on *mat*K+*trn*K.
Fig. S11. BI tree based on *psb*A +*trn*K.
Fig. S12. BI tree based on *psb*A+*rbc*L.
Fig. S13. BI tree based on *rbc*L+*trn*K.
Fig. S14. BI tree based on *mat*K+*psb*A+*rbc*L.
Fig. S15. BI tree based on *rbc*L+*trn*K+*psb*A.
Fig. S16. BI tree based on *rbc*L+*trn*K+*mat*K.
Fig. S17. BI tree based on *mat*K+*psb*A+*trn*K.
Fig. S18. BI tree based on ITS+*trn*K+*psb*A.
Fig. S19. BI tree based on ITS+*rbc*L+*trn*K.
Fig. S20. BI tree based on ITS+*psb*A+*rbc*L+*trn*K.
Fig. S21. BI tree based on ITS+*psb*A+*rbc*L.
Fig. S22. BI tree based on ITS+*mat*K+*psb*A.
Fig. S23. BI tree based on ITS+*mat*K+*rbc*L.
Fig. S24. BI tree based on ITS+*mat*K+*trn*K.
Fig. S25. BI tree based on ITS+*mat*K+*psb*A+*rbc*L.
Fig. S26. BI tree based on ITS+*mat*K+*psb*A+*trn*K.
Fig. S27. BI tree based on ITS+*mat*K+*trn*K+*rbc*L.
Fig. S28. BI tree based on ITS+*psb*A+*mat*K+*trn*K+*rbc*L.
Fig. S29. NJ tree based on ITS.
Fig. S30. NJ tree based on *trn*K.
Fig. S31. NJ tree based on *mat*K.
Fig. S32. NJ tree based on *psb*A
Fig. S33. NJ tree based on *rbc*L.
Fig. S34. NJ tree based on ITS+*mat*K.
Fig. S35. NJ tree based on ITS+*psb*A.
Fig. S36. NJ tree based on ITS+*rbc*L.
Fig. S37. NJ tree based on ITS+*trn*K.
Fig. S38. NJ tree based on *mat*K+*psb*A.
Fig. S39. NJ tree based on *mat*K+*rbc*L
Fig. S40. NJ tree based on *mat*K+*trn*K.
Fig. S41. NJ tree based on *psb*A+*rbc*L.
Fig. S42. NJ tree based on *psb*A+*trn*K.
Fig. S43. NJ tree based on *rbc*L+*trn*K.
Fig. S44. NJ tree based on ITS+*mat*K+*psb*A

Fig. S45. NJ tree based on ITS+*mat*K+*rbc*L.
Fig. S46. NJ tree based on ITS+*mat*K+*trn*K.
Fig. S47. NJ tree based on ITS+*rbc*L+*psb*A.
Fig. S48. NJ tree based on ITS+*rbc*L+*trn*K.
Fig. S49. NJ tree based on ITS+*trn*K+*psb*A.
Fig. S50. NJ tree based on *mat*K+*psb*A+*rbc*L.
Fig. S51. NJ tree based on *mat*K+*psb*A+*trn*K
Fig. S52. NJ tree based on *rbc*L+*trn*K+*mat*K.
Fig. S53. NJ tree based on *rbc*L+*trn*K+*psb*A.
Fig. S54. NJ tree based on ITS+*mat*K+*psb*A+*rbc*L.
Fig. S55. NJ tree based on ITS+*mat*K+*psb*A+*trn*K.
Fig. S56. NJ tree based on ITS+*mat*K+*rbc*L+*trn*K.
Fig. S57. NJ tree based on ITS+*rbc*L+*trn*K+*psb*A.
Fig. S58. NJ tree based on *rbc*L+*trn*K+*psb*A+*mat*K
Fig. S59. NJ tree based on ITS+*psb*A+*mat*K+*trn*K+*rbc*L.
