# Supplementary figures and images for: Identification of species and materia medica within Saussurea subg. Amphilaena based on DNA barcodes
Source: PeerJ. 2019 Feb 1;7:e6357. doi: 10.7717/peerj.6357 (PMC6361007; doi:10.7717/peerj.6357)

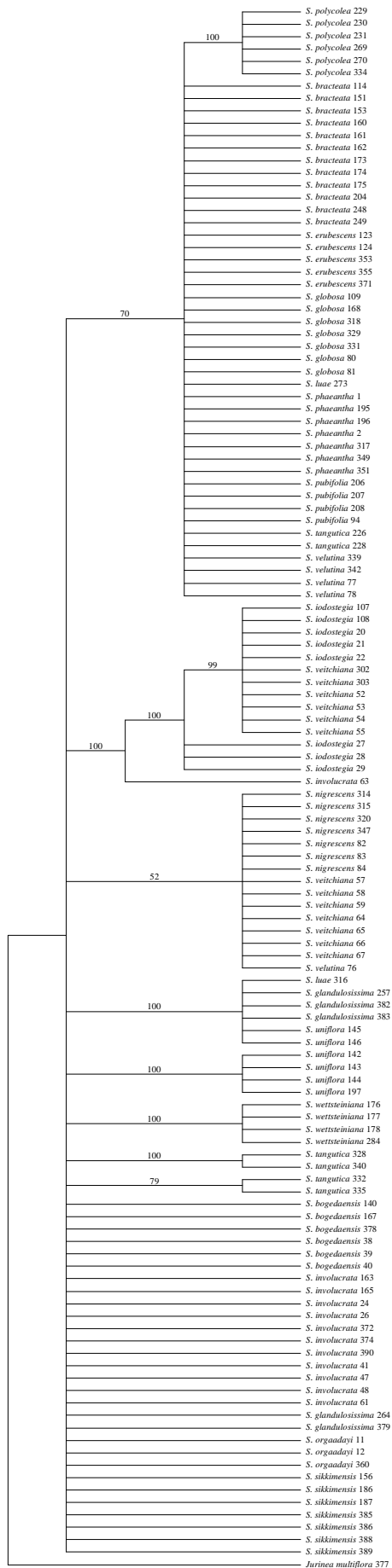

Supplement: Supplemental Information 1 [file peerj-07-6357-s001.zip › Supporting Information/Fig. S1.pdf]

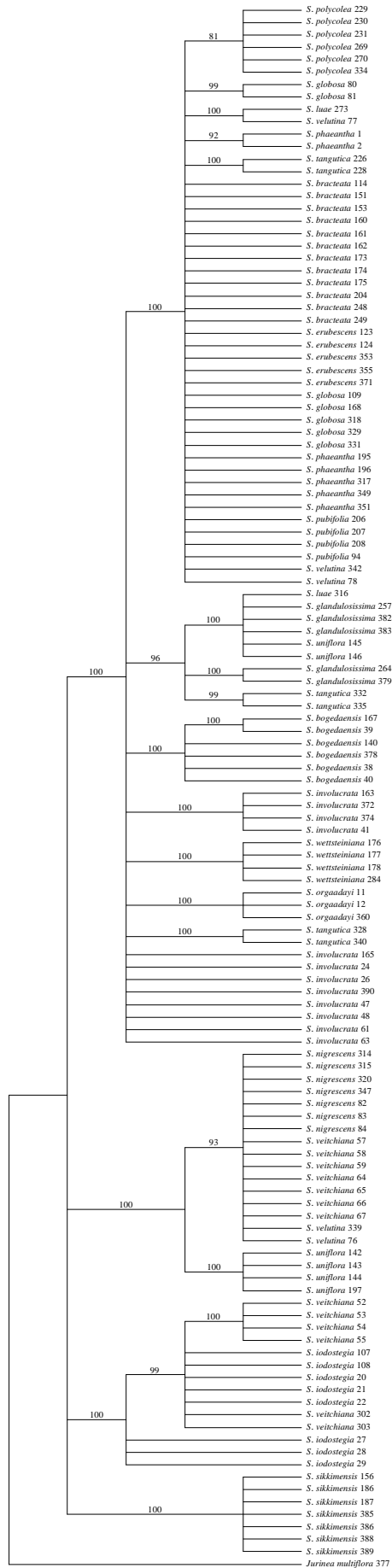

Supplement: Supplemental Information 1 [file peerj-07-6357-s001.zip › Supporting Information/Fig. S10.pdf]

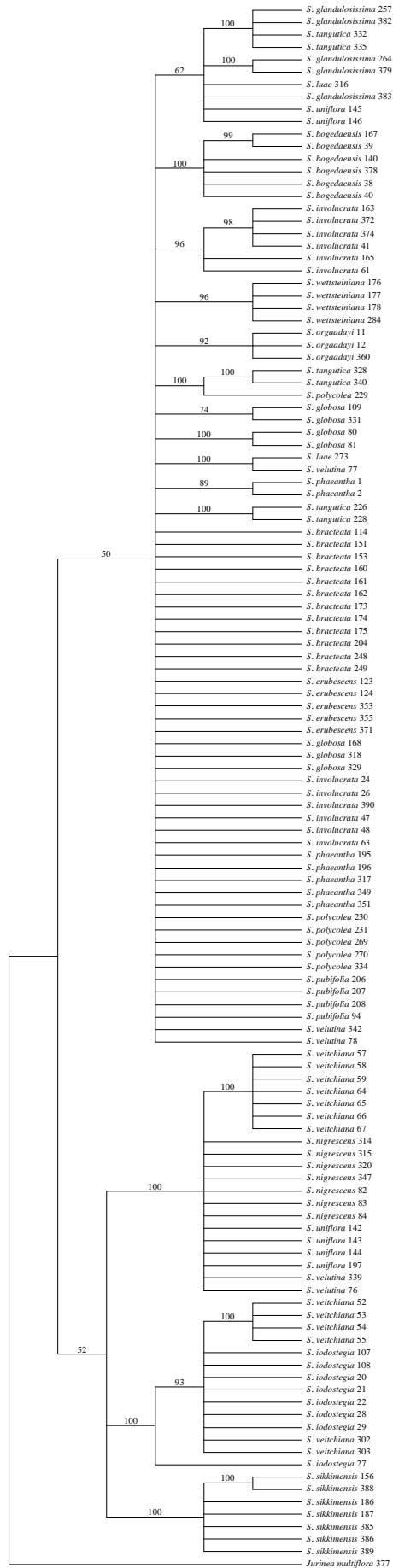

Supplement: Supplemental Information 1 [file peerj-07-6357-s001.zip › Supporting Information/Fig. S11.pdf]

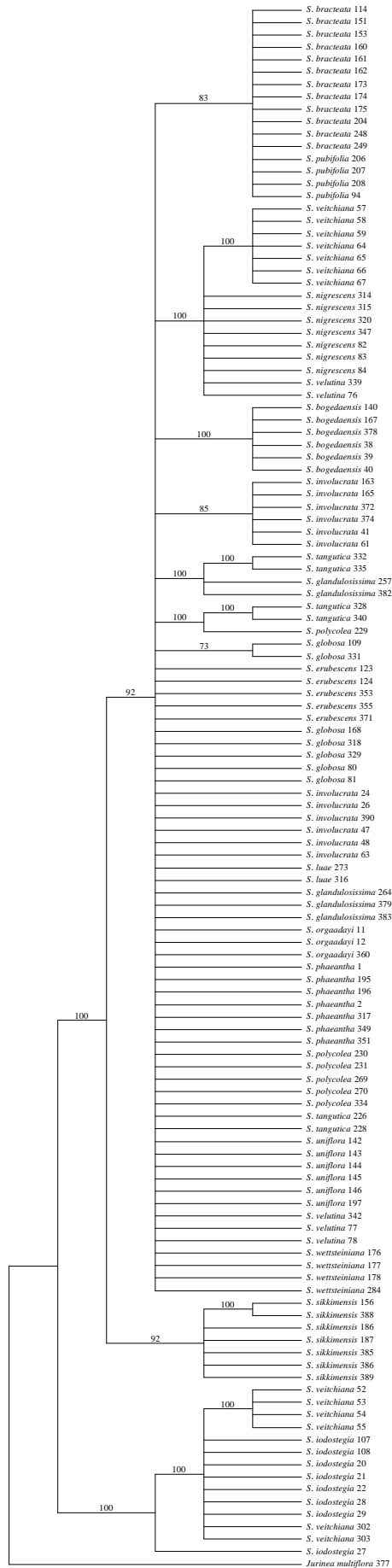

Supplement: Supplemental Information 1 [file peerj-07-6357-s001.zip › Supporting Information/Fig. S12.pdf]

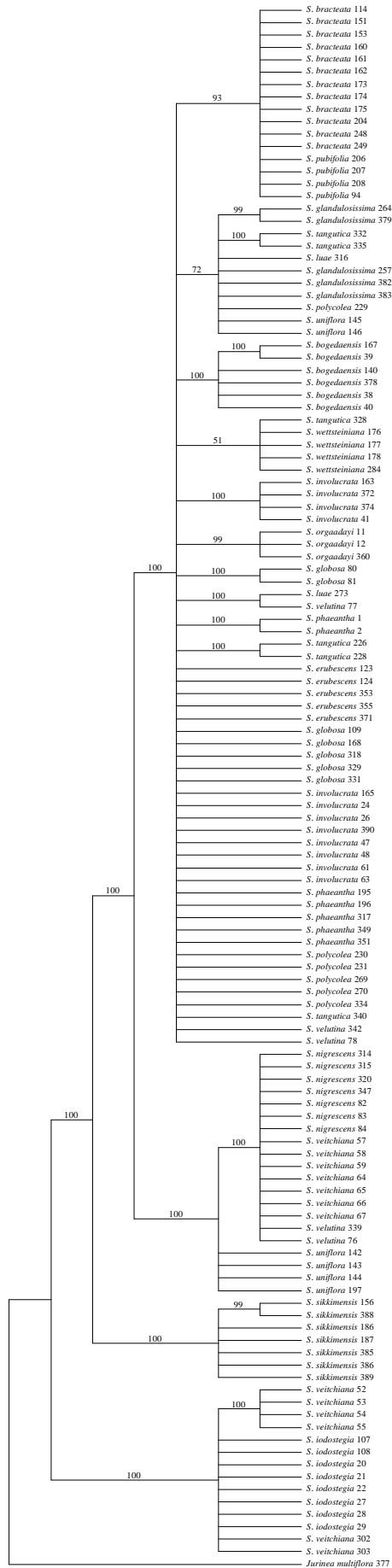

Supplement: Supplemental Information 1 [file peerj-07-6357-s001.zip › Supporting Information/Fig. S13.pdf]

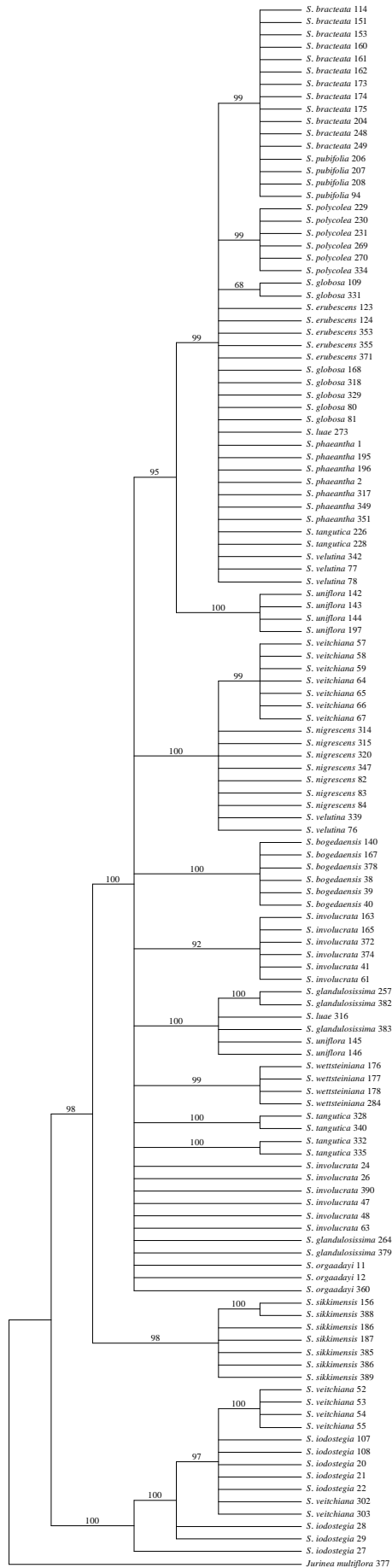

Supplement: Supplemental Information 1 [file peerj-07-6357-s001.zip › Supporting Information/Fig. S14.pdf]

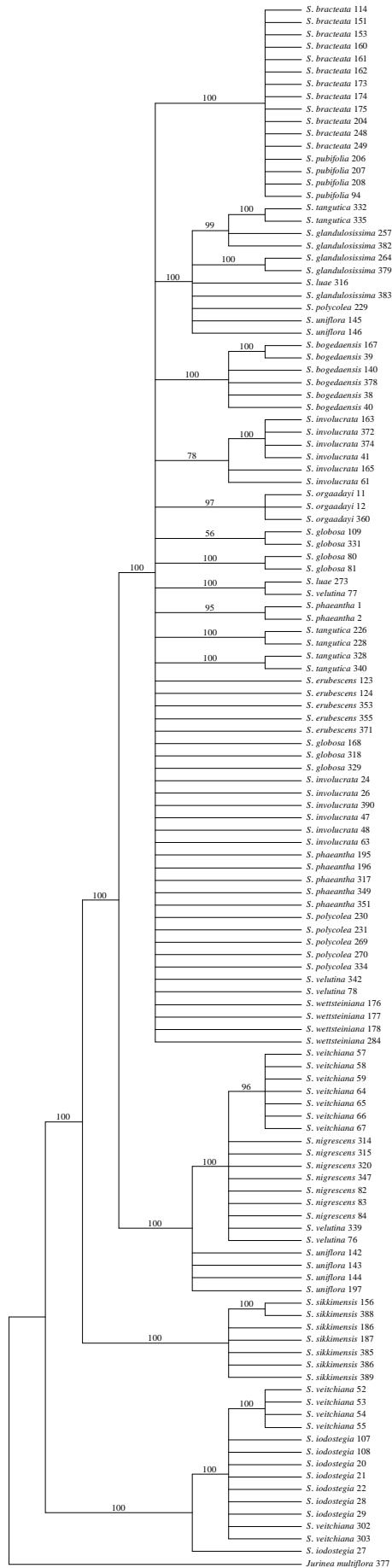

Supplement: Supplemental Information 1 [file peerj-07-6357-s001.zip › Supporting Information/Fig. S15.pdf]

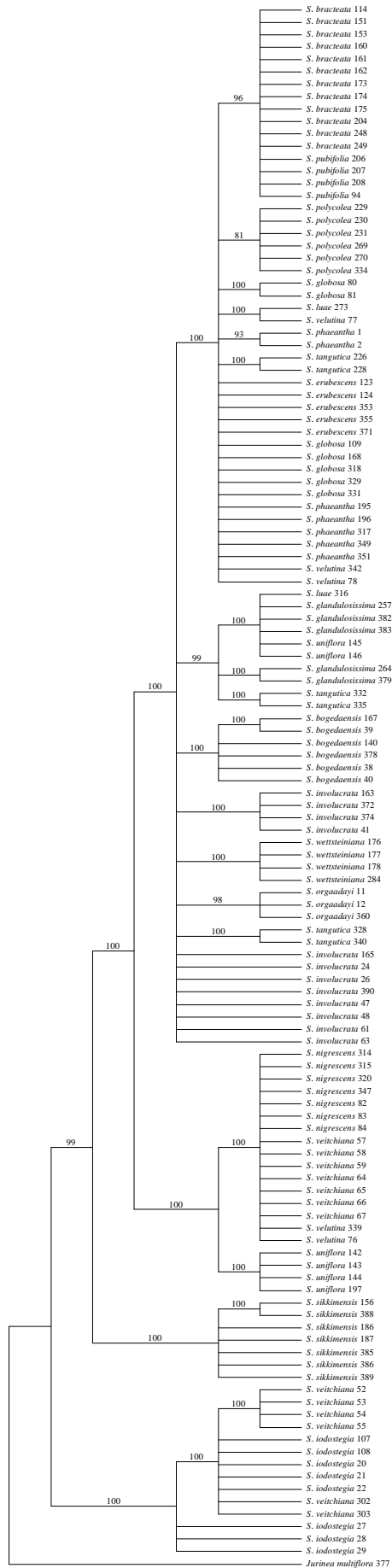

Supplement: Supplemental Information 1 [file peerj-07-6357-s001.zip › Supporting Information/Fig. S16.pdf]

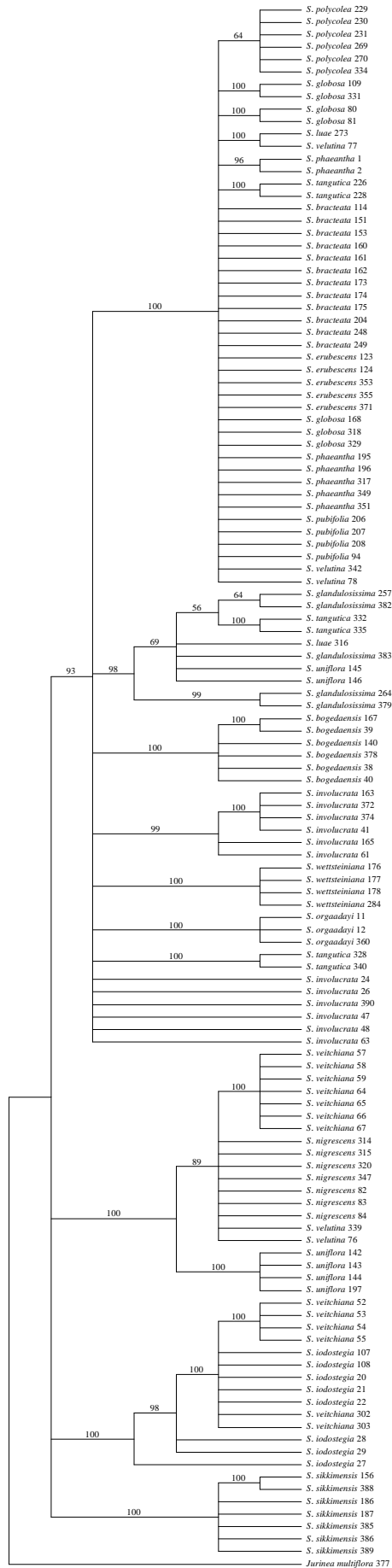

Supplement: Supplemental Information 1 [file peerj-07-6357-s001.zip › Supporting Information/Fig. S17.pdf]

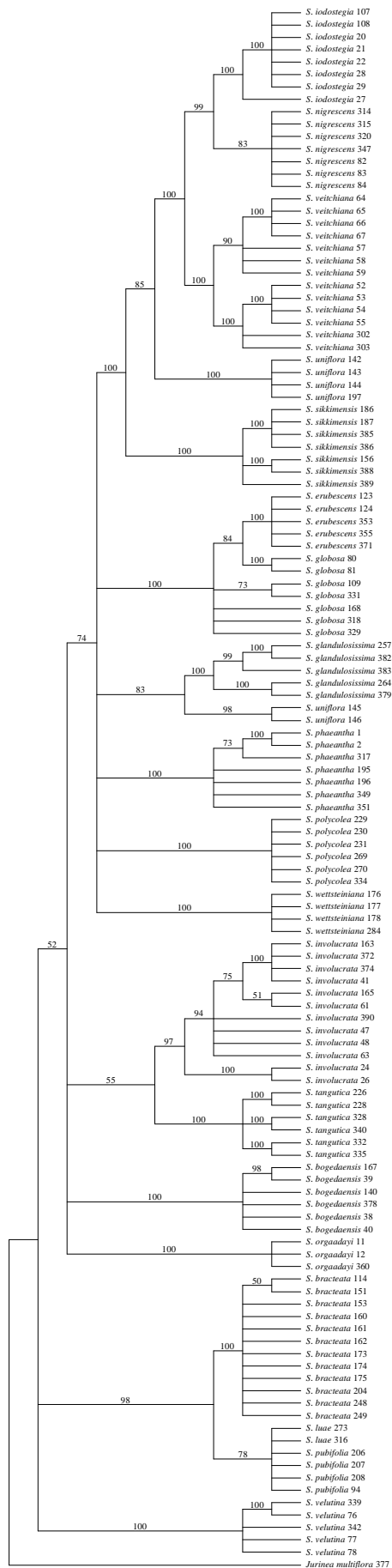

Supplement: Supplemental Information 1 [file peerj-07-6357-s001.zip › Supporting Information/Fig. S18.pdf]

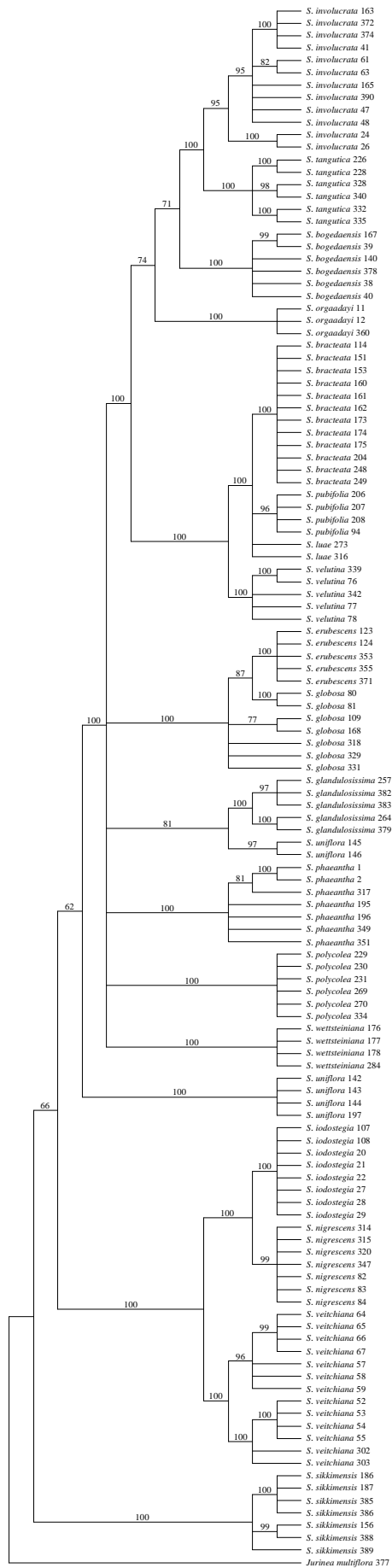

Supplement: Supplemental Information 1 [file peerj-07-6357-s001.zip › Supporting Information/Fig. S19.pdf]

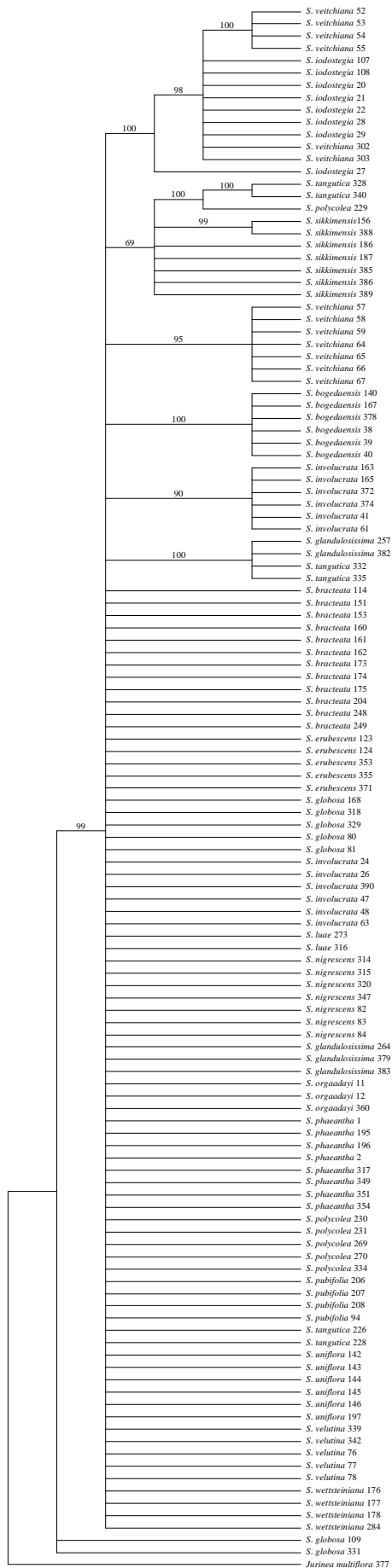

Supplement: Supplemental Information 1 [file peerj-07-6357-s001.zip › Supporting Information/Fig. S2.pdf]

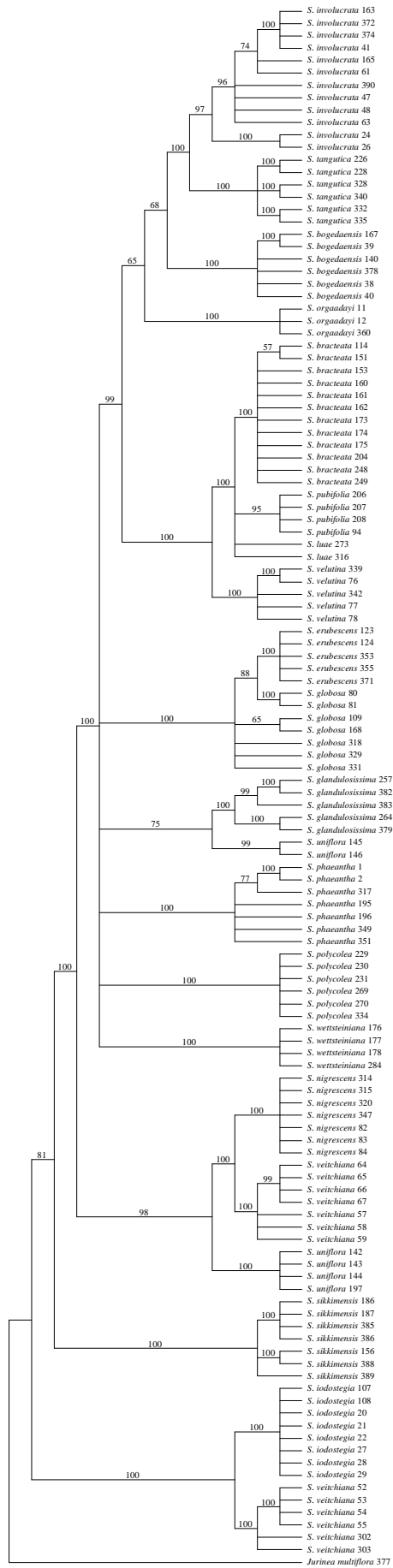

Supplement: Supplemental Information 1 [file peerj-07-6357-s001.zip › Supporting Information/Fig. S20.pdf]

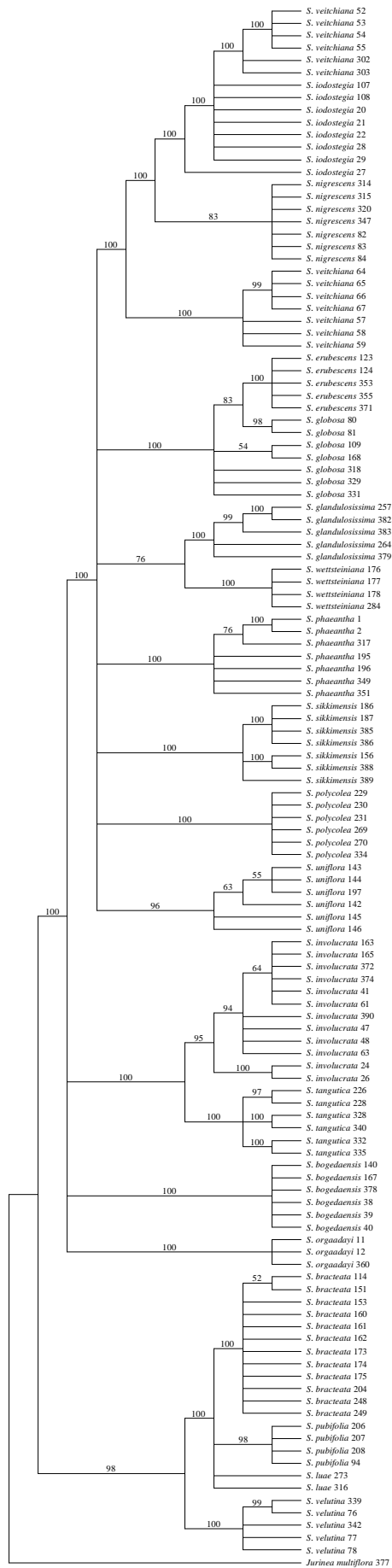

Supplement: Supplemental Information 1 [file peerj-07-6357-s001.zip › Supporting Information/Fig. S21.pdf]

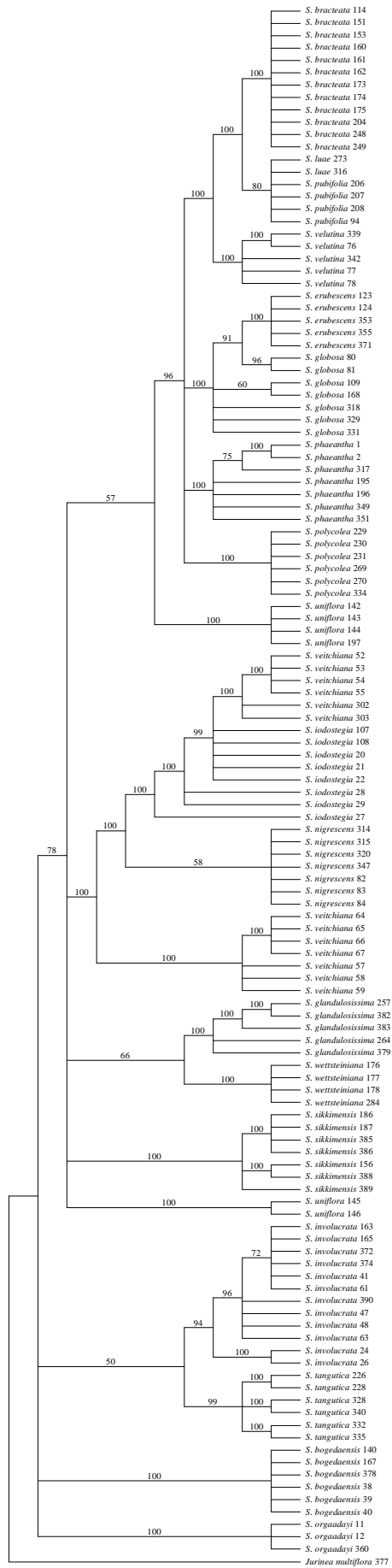

Supplement: Supplemental Information 1 [file peerj-07-6357-s001.zip › Supporting Information/Fig. S22.pdf]

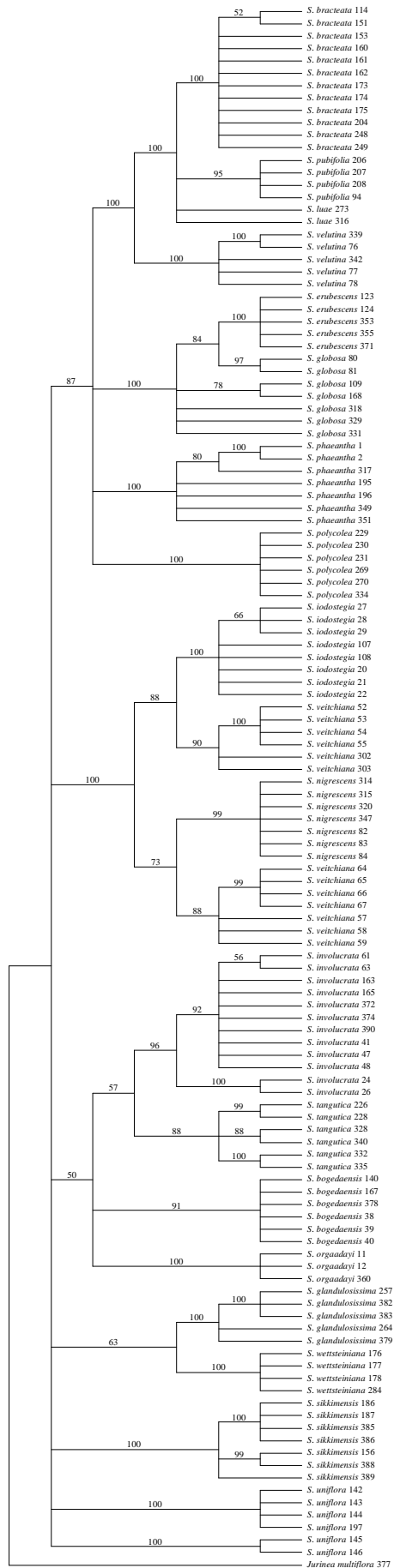

Supplement: Supplemental Information 1 [file peerj-07-6357-s001.zip › Supporting Information/Fig. S23.pdf]

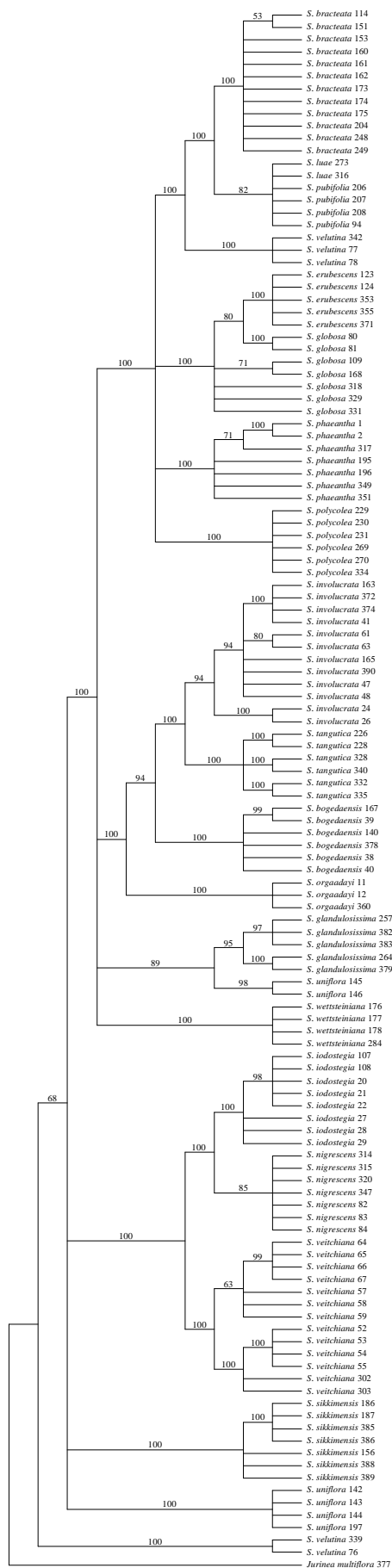

Supplement: Supplemental Information 1 [file peerj-07-6357-s001.zip › Supporting Information/Fig. S24.pdf]

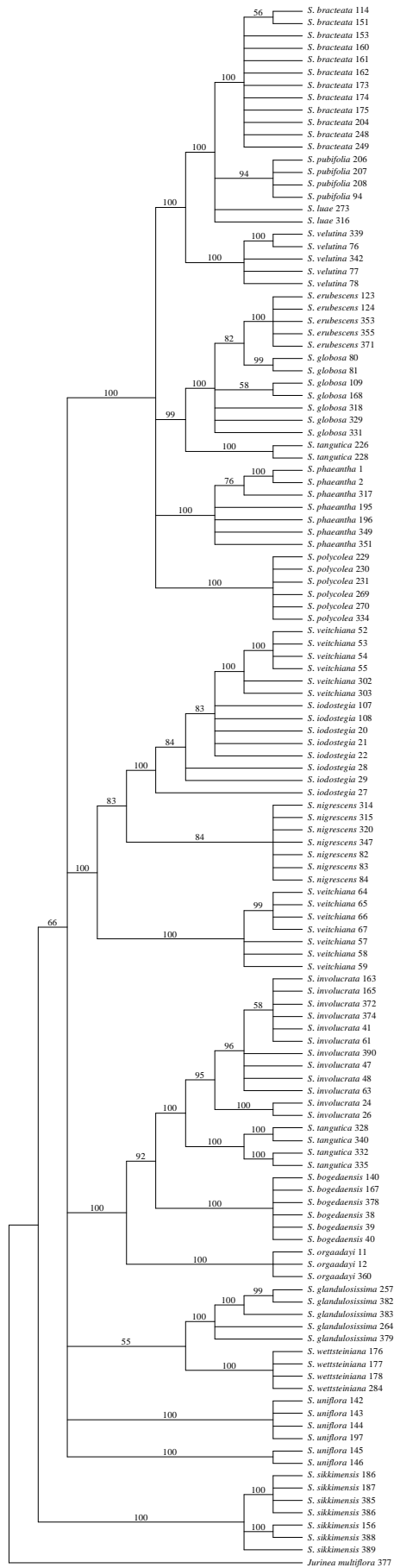

Supplement: Supplemental Information 1 [file peerj-07-6357-s001.zip › Supporting Information/Fig. S25.pdf]

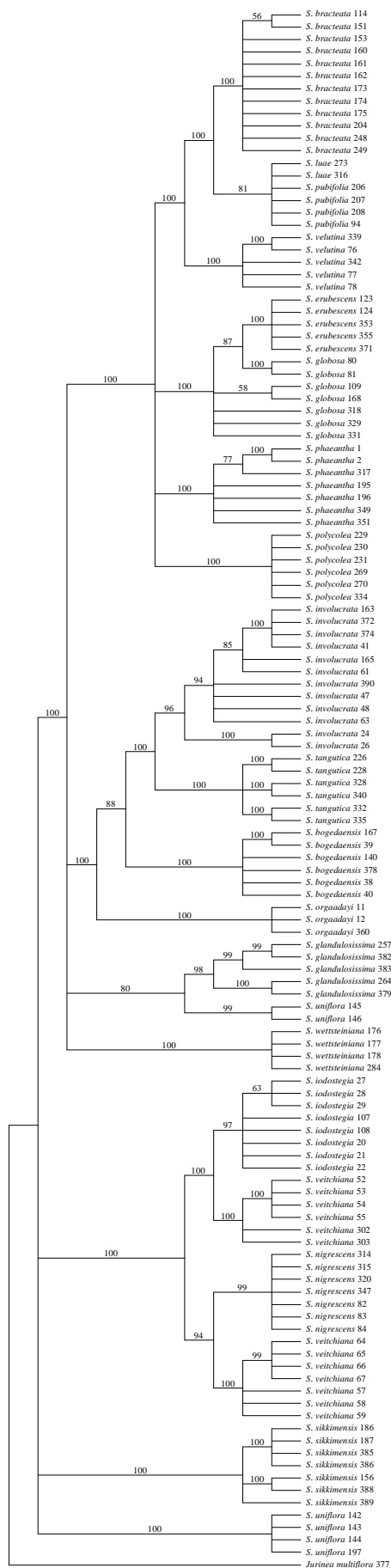

Supplement: Supplemental Information 1 [file peerj-07-6357-s001.zip › Supporting Information/Fig. S26.pdf]

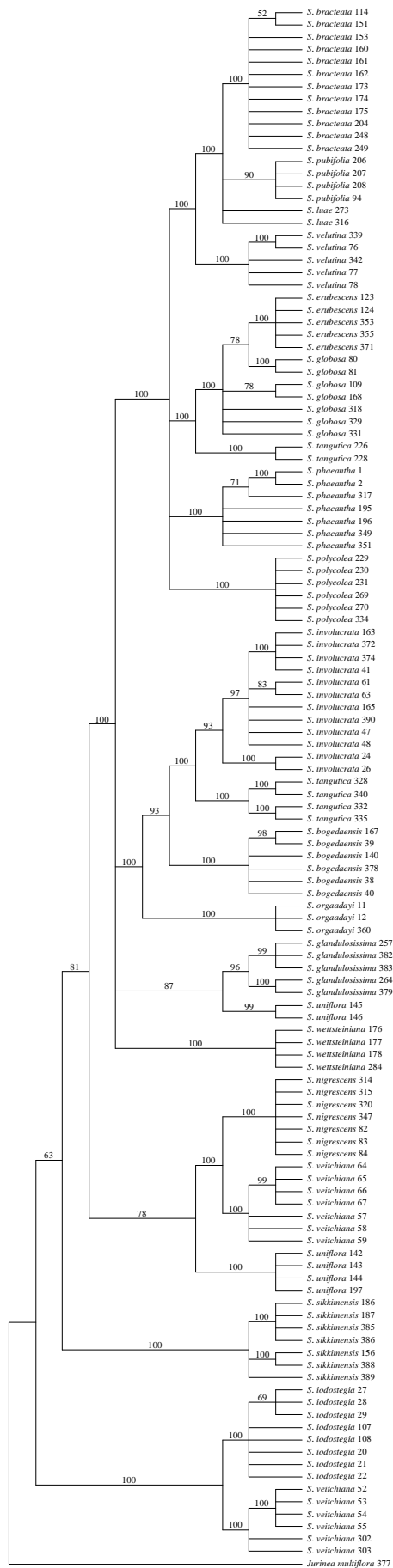

Supplement: Supplemental Information 1 [file peerj-07-6357-s001.zip › Supporting Information/Fig. S27.pdf]

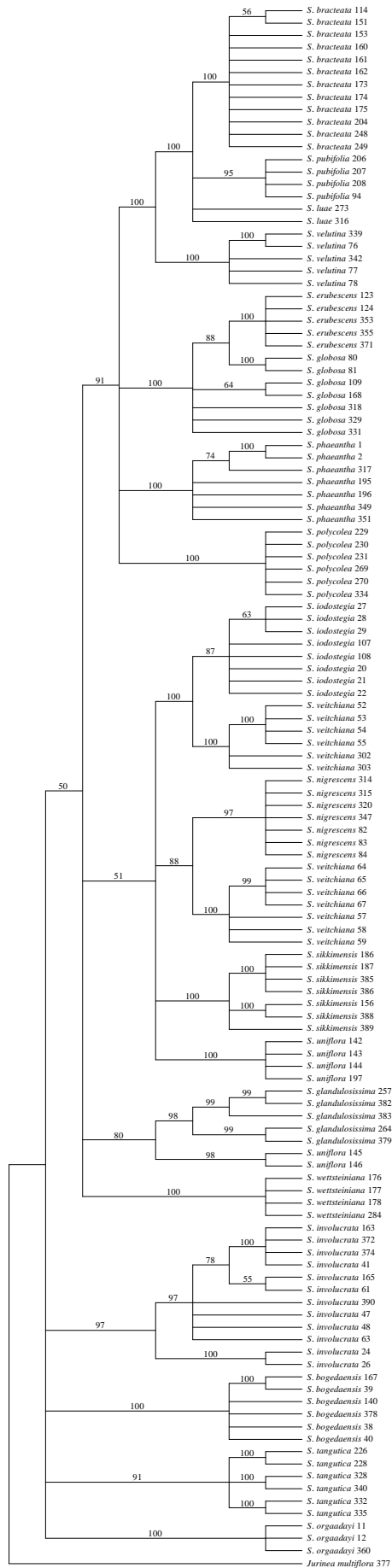

Supplement: Supplemental Information 1 [file peerj-07-6357-s001.zip › Supporting Information/Fig. S28.pdf]

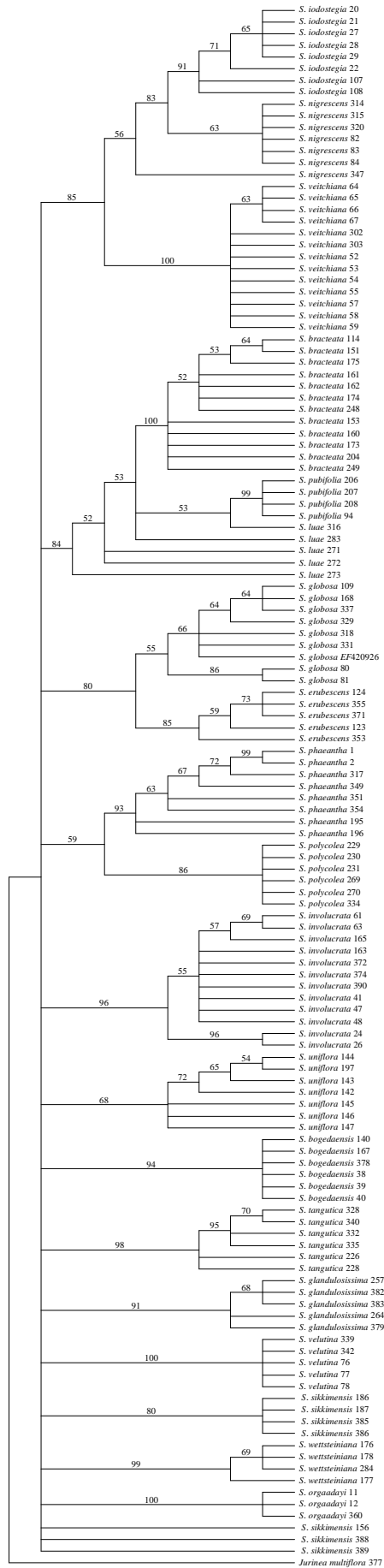

Supplement: Supplemental Information 1 [file peerj-07-6357-s001.zip › Supporting Information/Fig. S29.pdf]

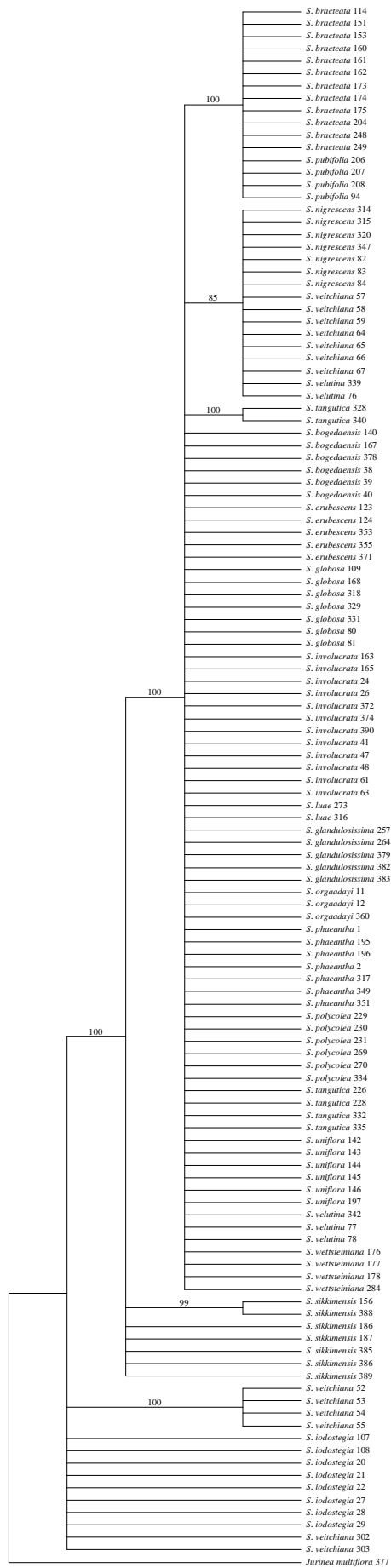

Supplement: Supplemental Information 1 [file peerj-07-6357-s001.zip › Supporting Information/Fig. S3.pdf]

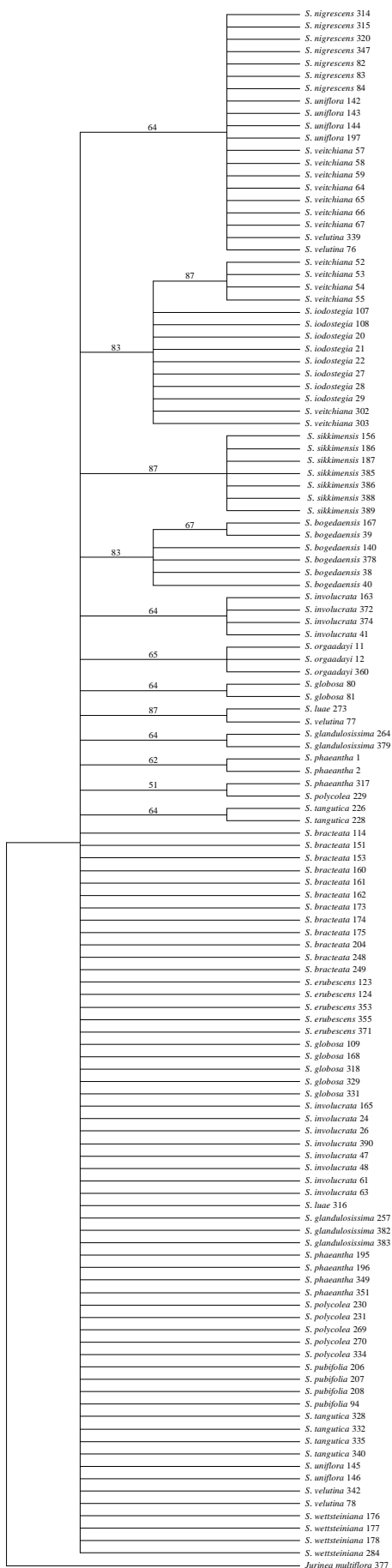

Supplement: Supplemental Information 1 [file peerj-07-6357-s001.zip › Supporting Information/Fig. S30.pdf]

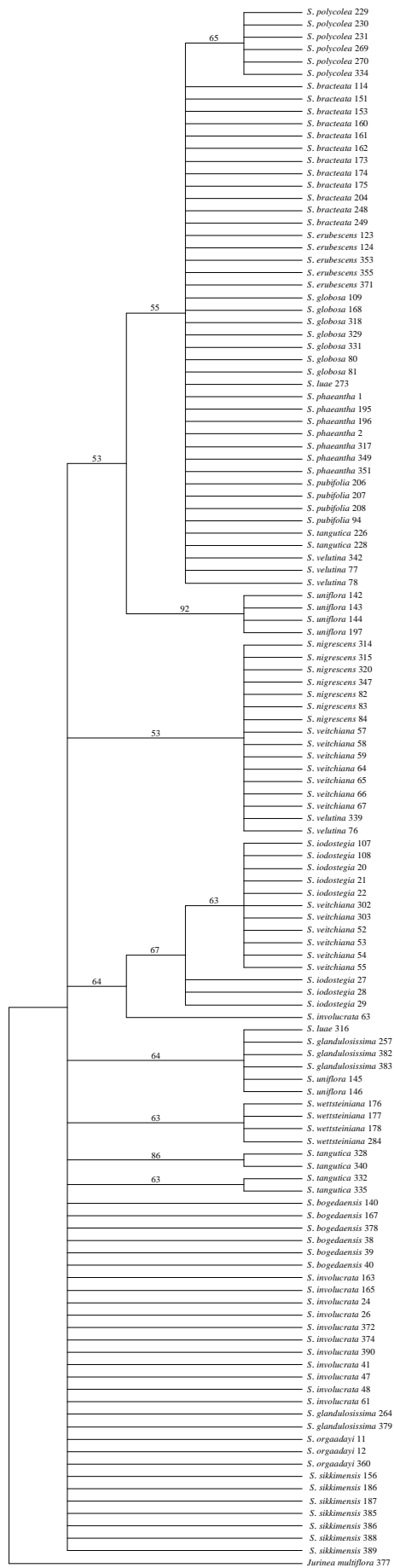

Supplement: Supplemental Information 1 [file peerj-07-6357-s001.zip › Supporting Information/Fig. S31.pdf]

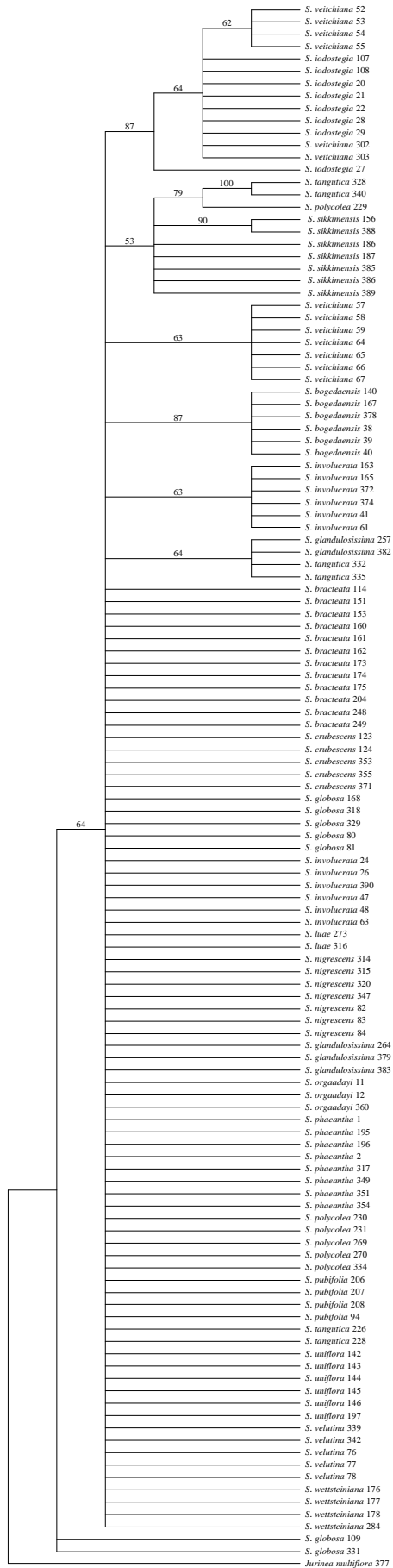

Supplement: Supplemental Information 1 [file peerj-07-6357-s001.zip › Supporting Information/Fig. S32.pdf]

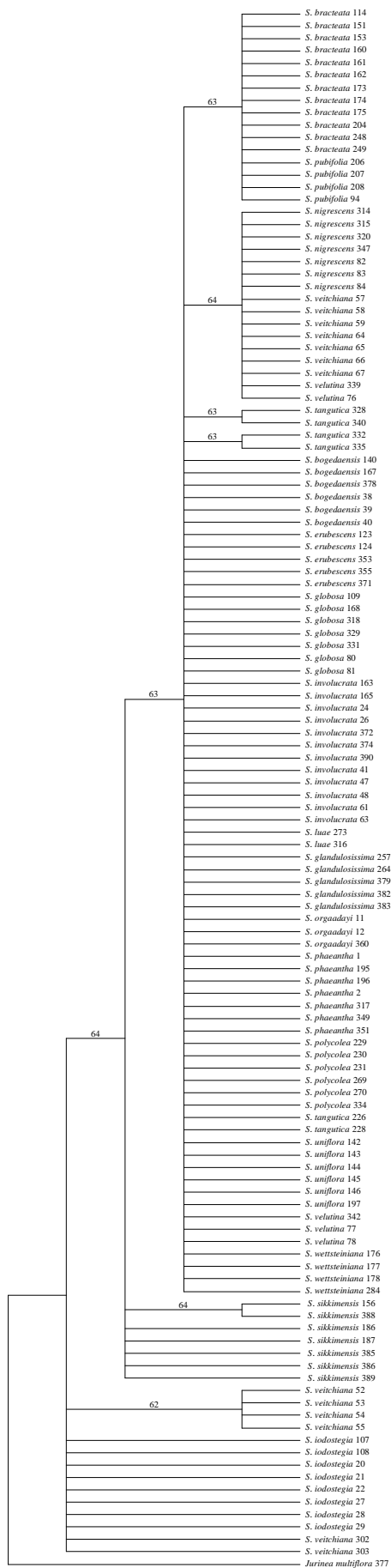

Supplement: Supplemental Information 1 [file peerj-07-6357-s001.zip › Supporting Information/Fig. S33.pdf]

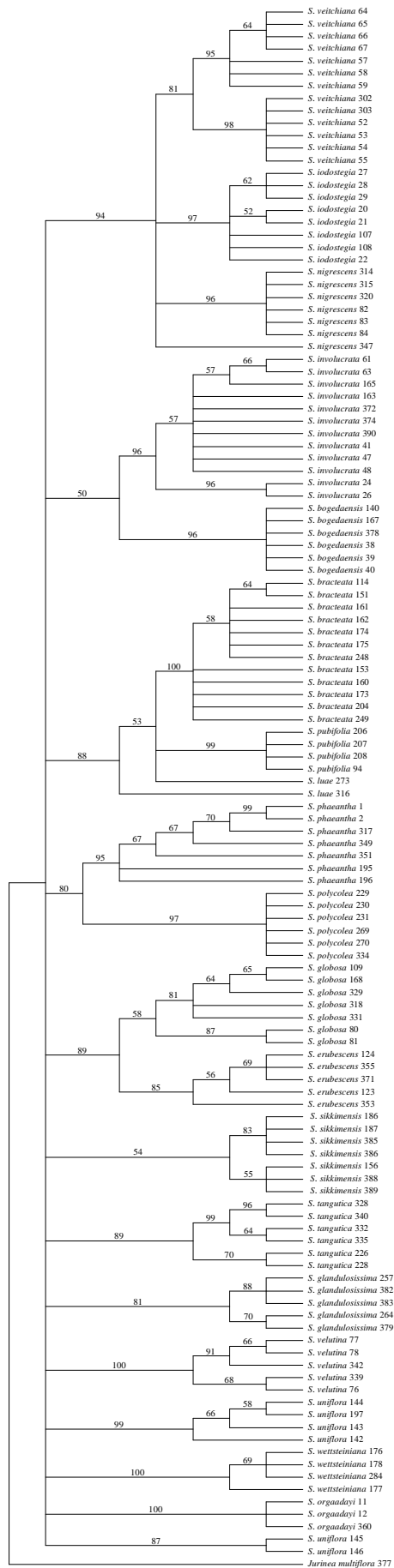

Supplement: Supplemental Information 1 [file peerj-07-6357-s001.zip › Supporting Information/Fig. S34.pdf]

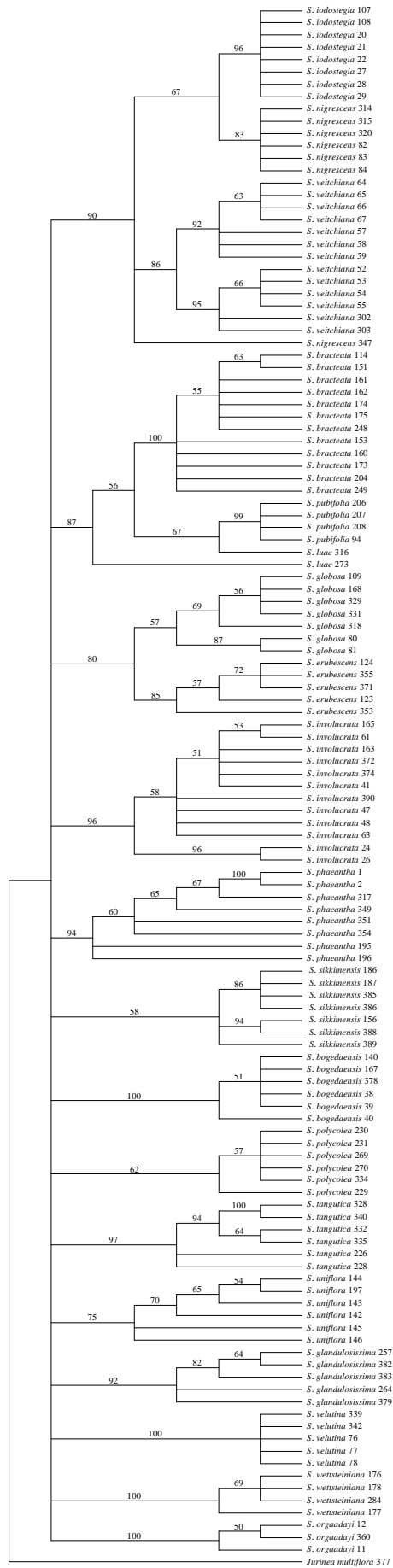

Supplement: Supplemental Information 1 [file peerj-07-6357-s001.zip › Supporting Information/Fig. S35.pdf]

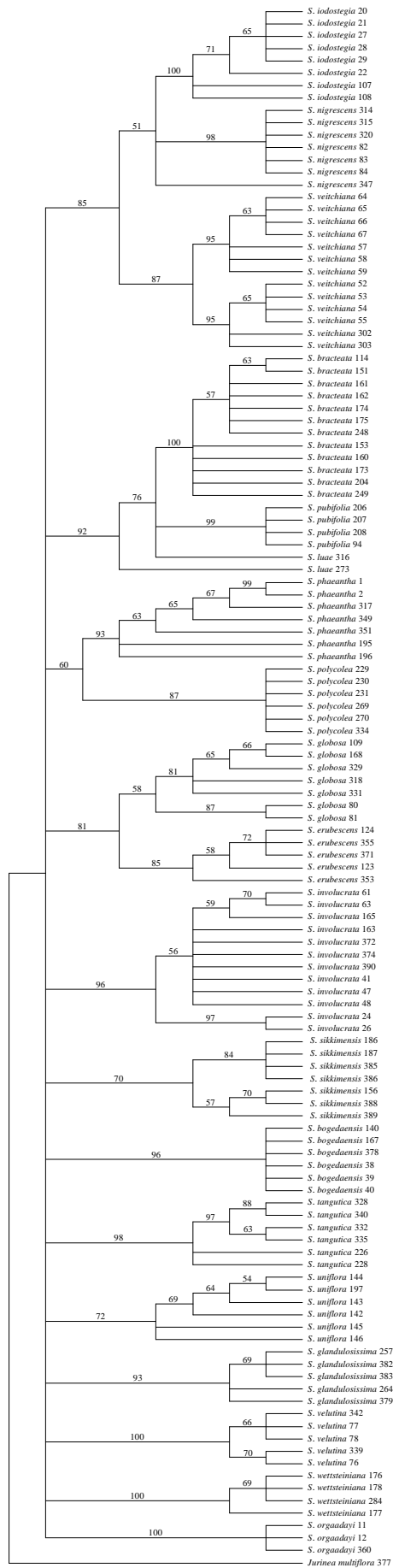

Supplement: Supplemental Information 1 [file peerj-07-6357-s001.zip › Supporting Information/Fig. S36.pdf]

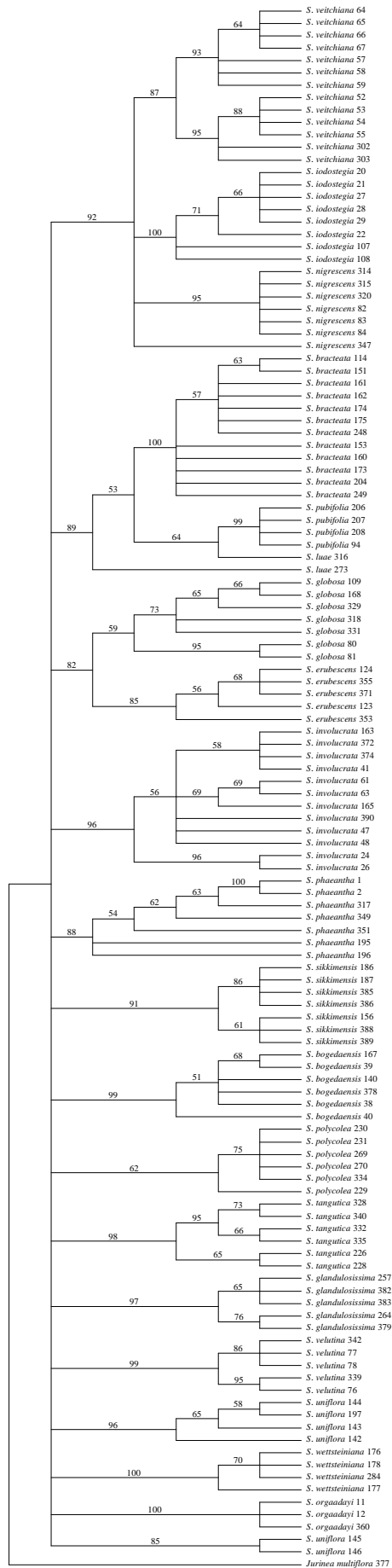

Supplement: Supplemental Information 1 [file peerj-07-6357-s001.zip › Supporting Information/Fig. S37.pdf]

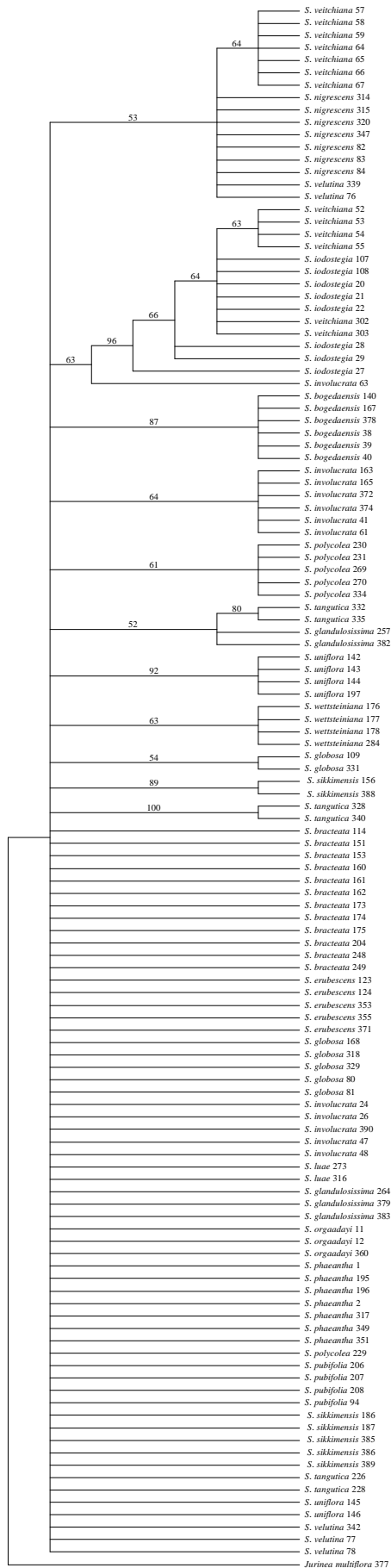

Supplement: Supplemental Information 1 [file peerj-07-6357-s001.zip › Supporting Information/Fig. S38.pdf]

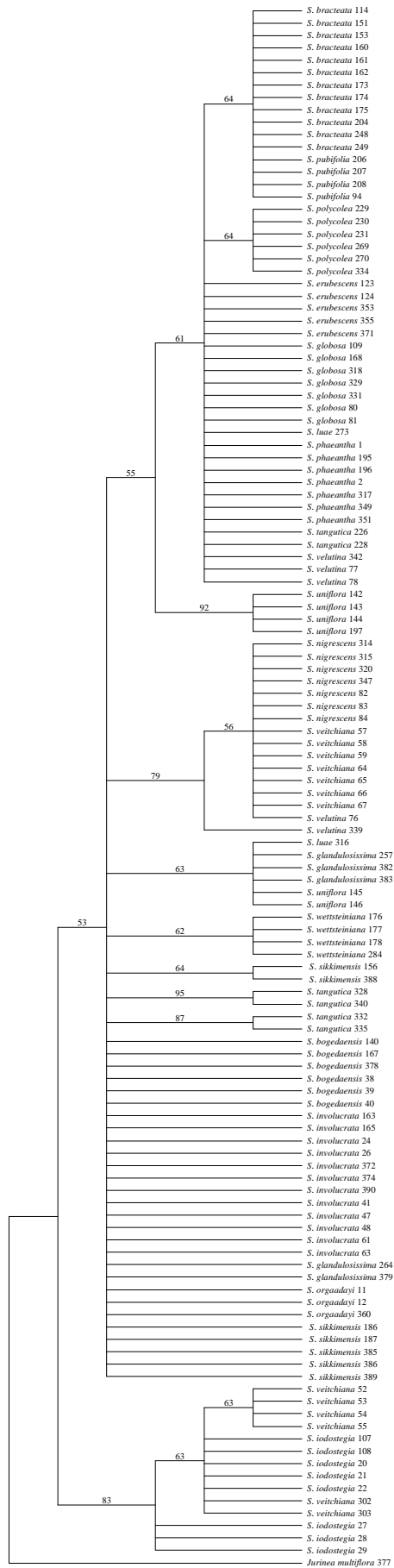

Supplement: Supplemental Information 1 [file peerj-07-6357-s001.zip › Supporting Information/Fig. S39.pdf]

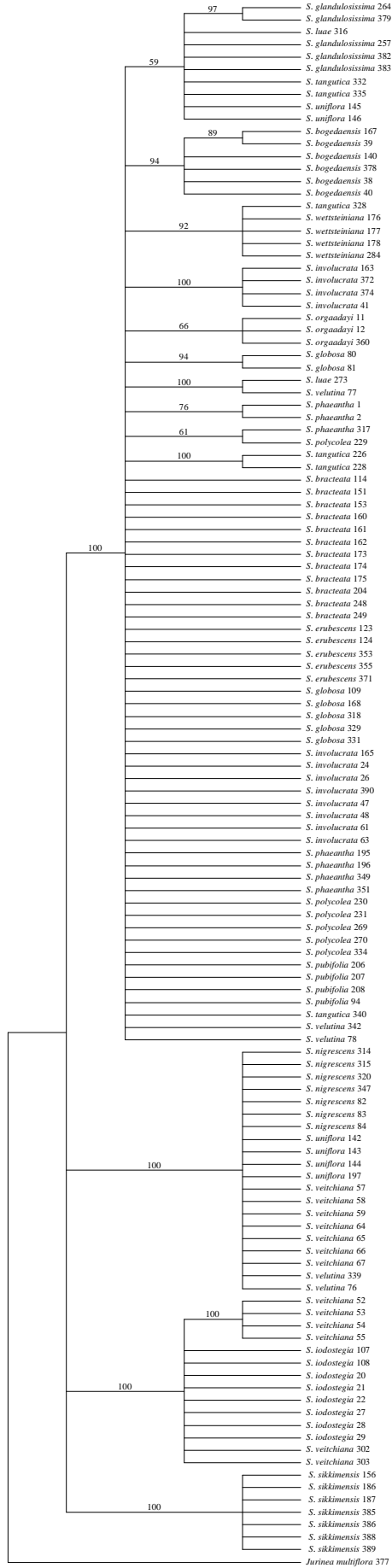

Supplement: Supplemental Information 1 [file peerj-07-6357-s001.zip › Supporting Information/Fig. S4.pdf]

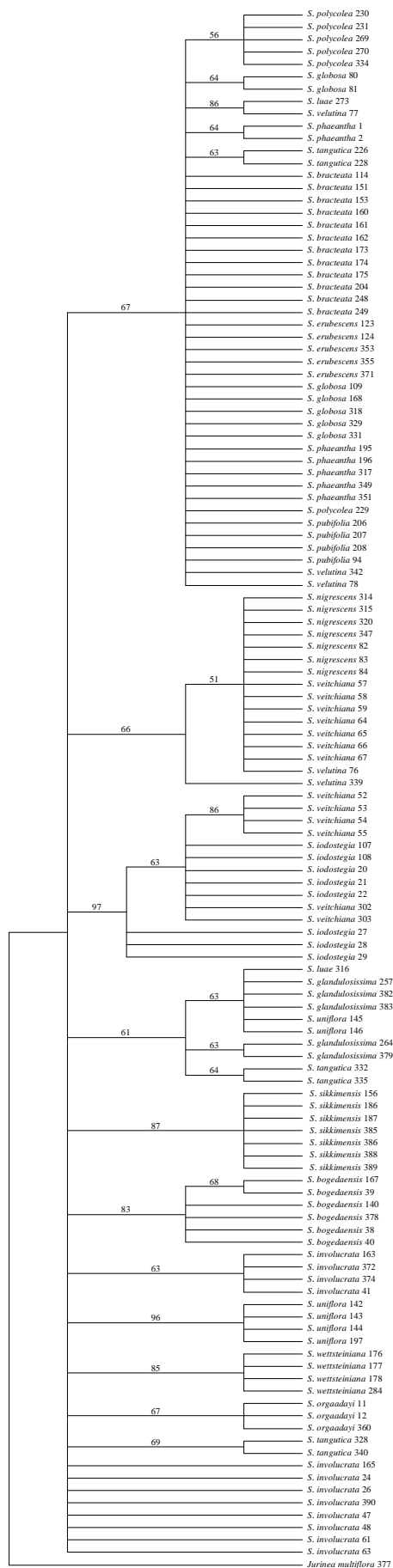

Supplement: Supplemental Information 1 [file peerj-07-6357-s001.zip › Supporting Information/Fig. S40.pdf]

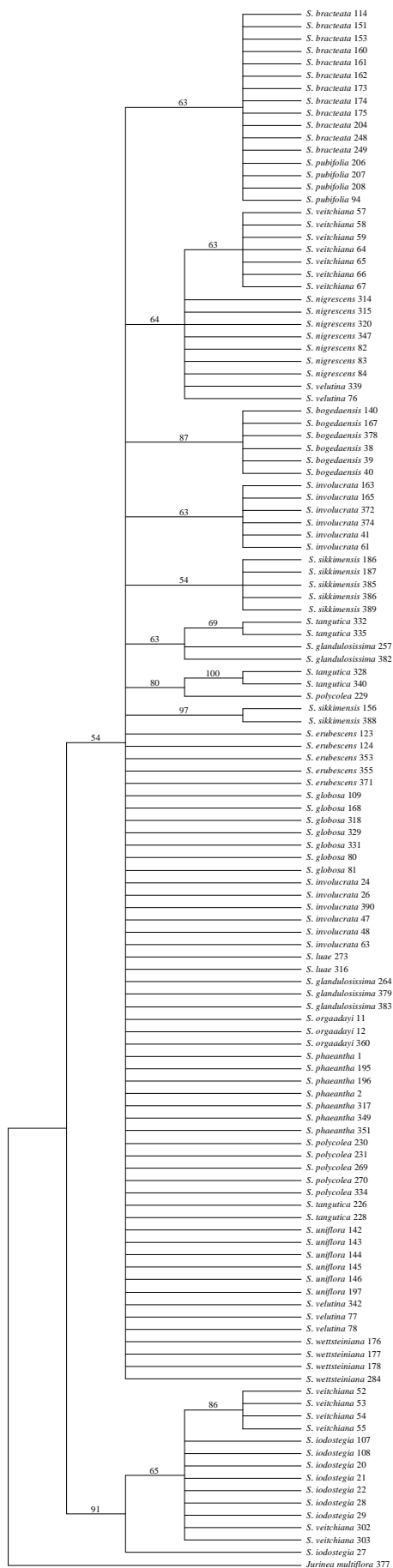

Supplement: Supplemental Information 1 [file peerj-07-6357-s001.zip › Supporting Information/Fig. S41.pdf]

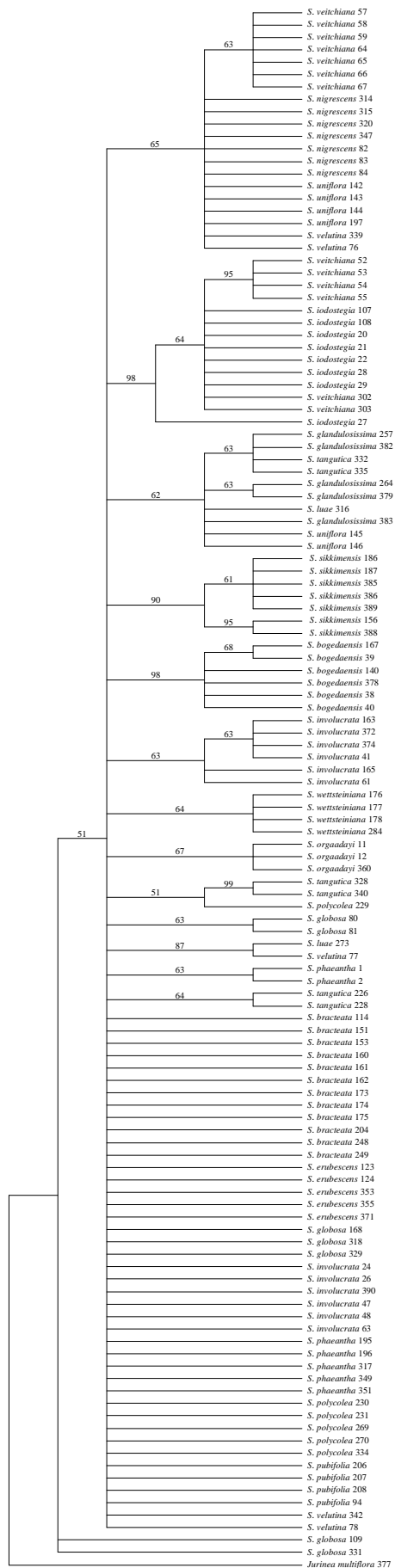

Supplement: Supplemental Information 1 [file peerj-07-6357-s001.zip › Supporting Information/Fig. S42.pdf]

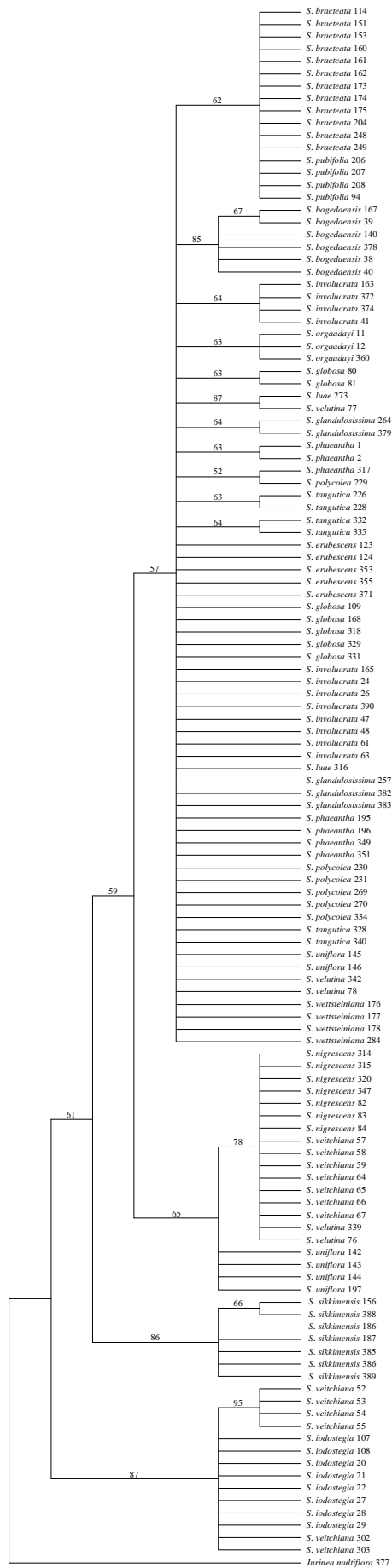

Supplement: Supplemental Information 1 [file peerj-07-6357-s001.zip › Supporting Information/Fig. S43.pdf]

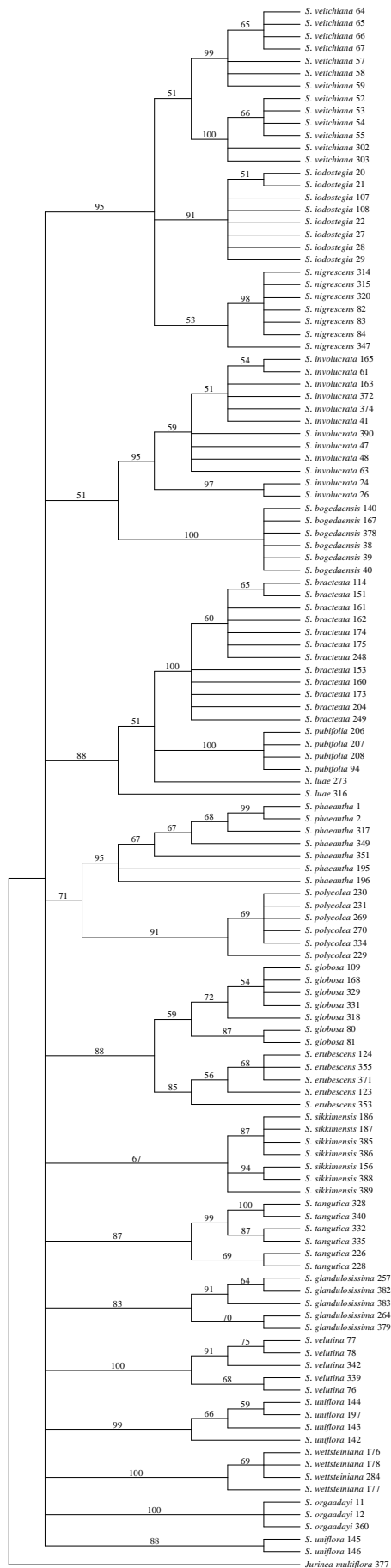

Supplement: Supplemental Information 1 [file peerj-07-6357-s001.zip › Supporting Information/Fig. S44.pdf]

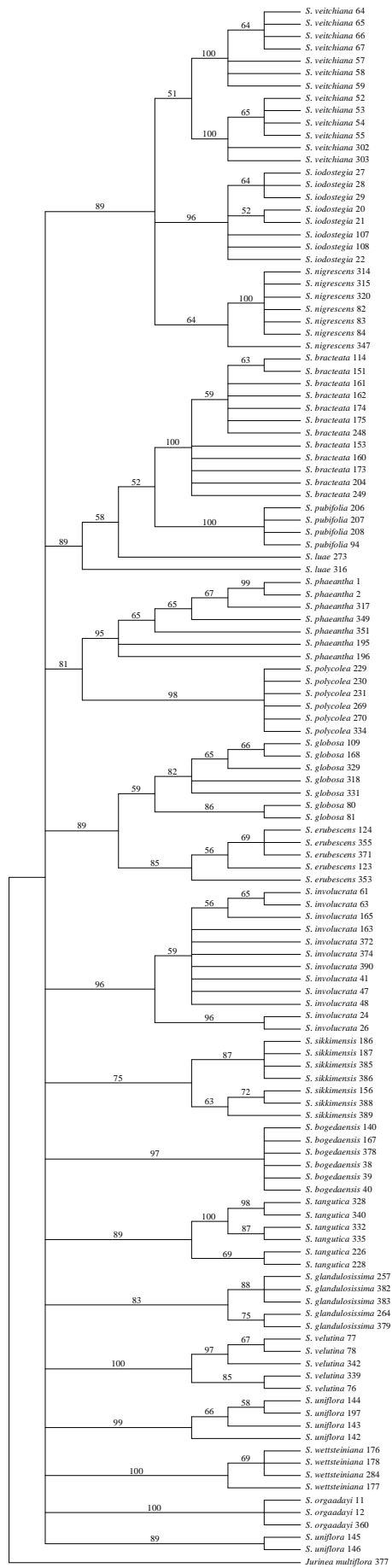

Supplement: Supplemental Information 1 [file peerj-07-6357-s001.zip › Supporting Information/Fig. S45.pdf]

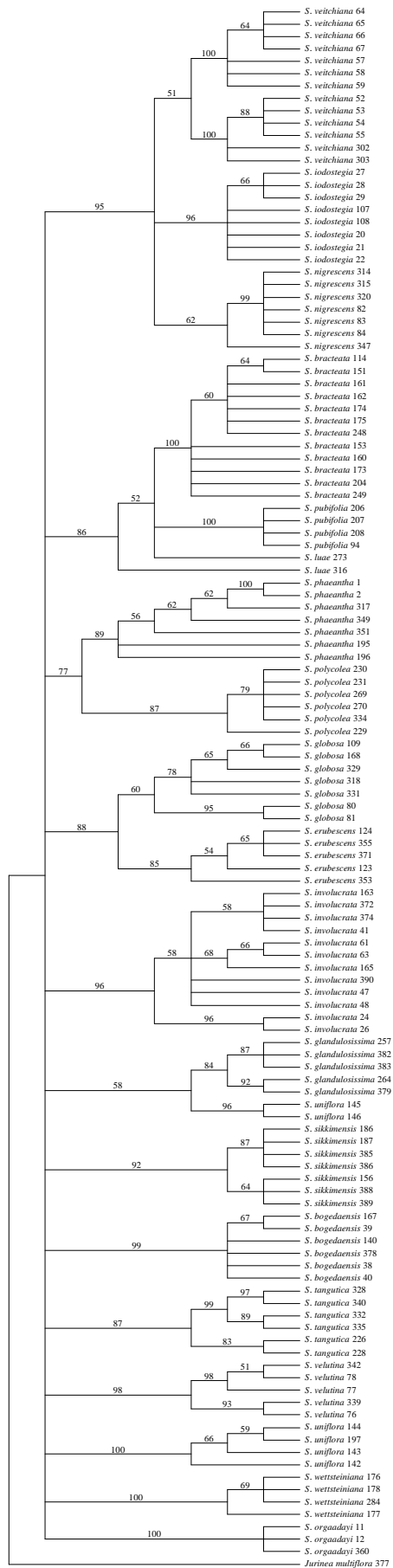

Supplement: Supplemental Information 1 [file peerj-07-6357-s001.zip › Supporting Information/Fig. S46.pdf]

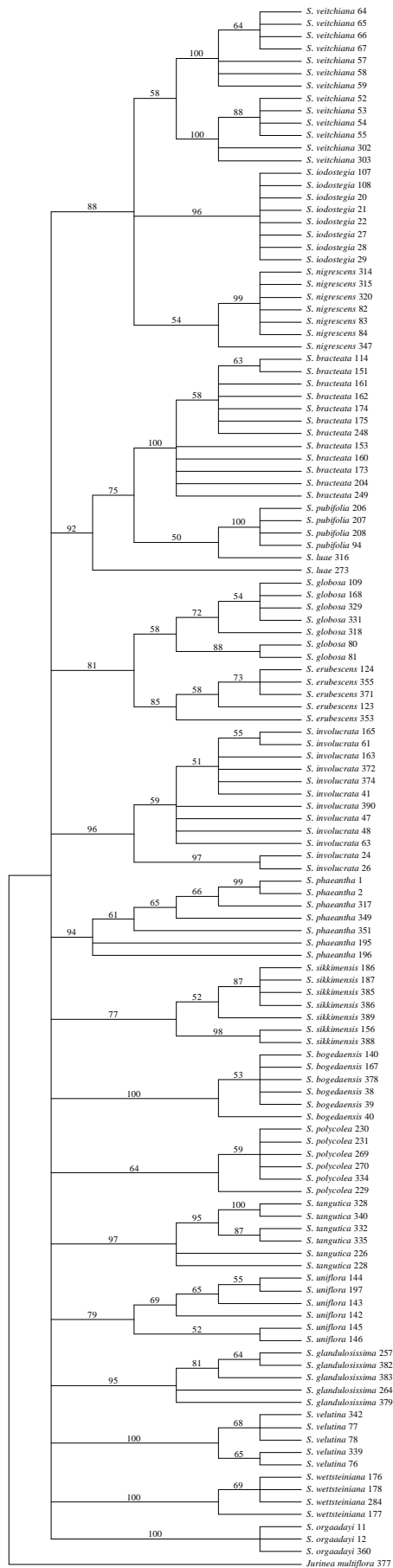

Supplement: Supplemental Information 1 [file peerj-07-6357-s001.zip › Supporting Information/Fig. S47.pdf]

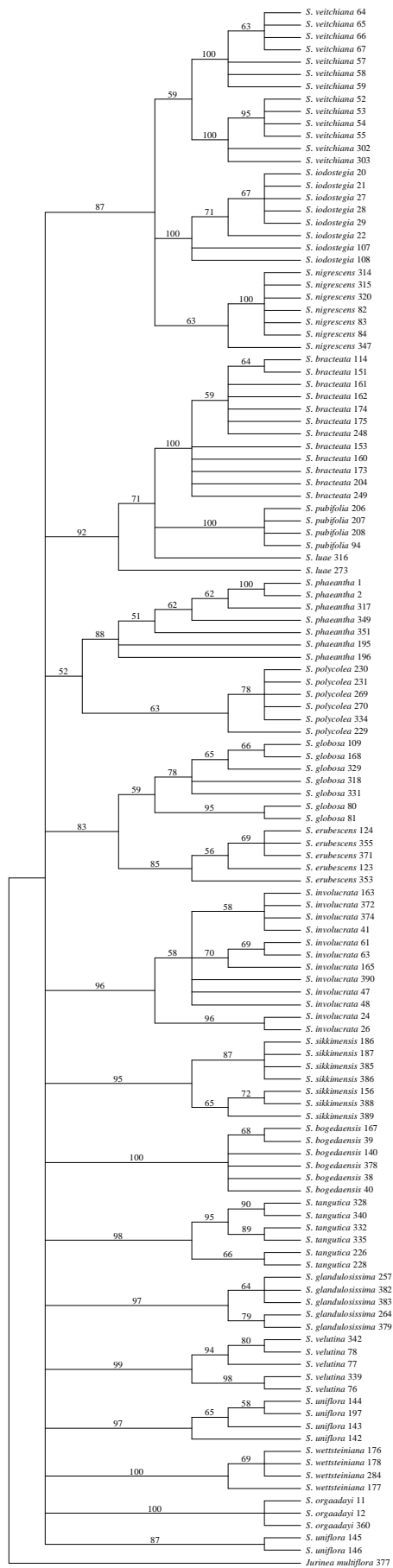

Supplement: Supplemental Information 1 [file peerj-07-6357-s001.zip › Supporting Information/Fig. S48.pdf]

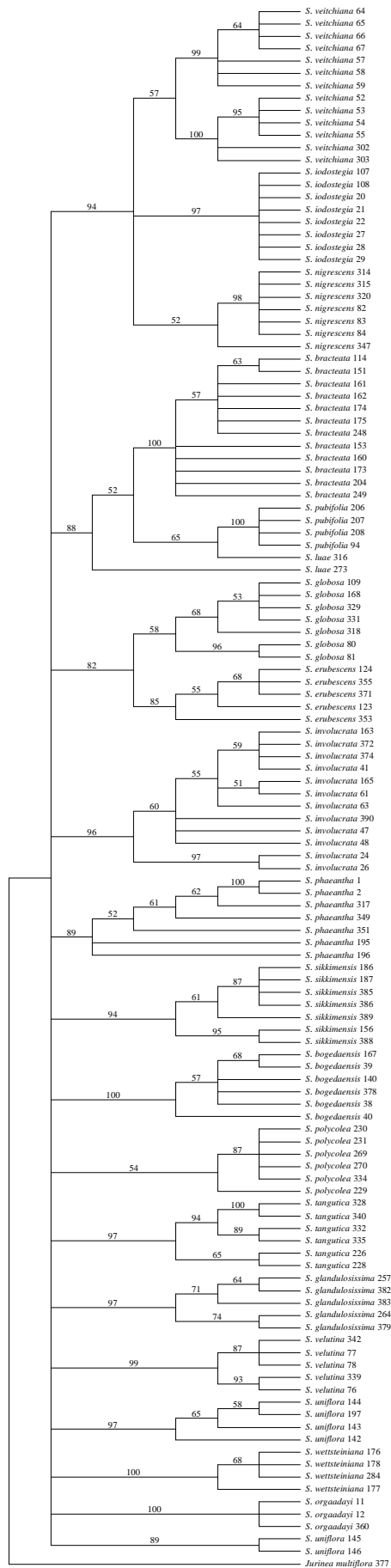

Supplement: Supplemental Information 1 [file peerj-07-6357-s001.zip › Supporting Information/Fig. S49.pdf]

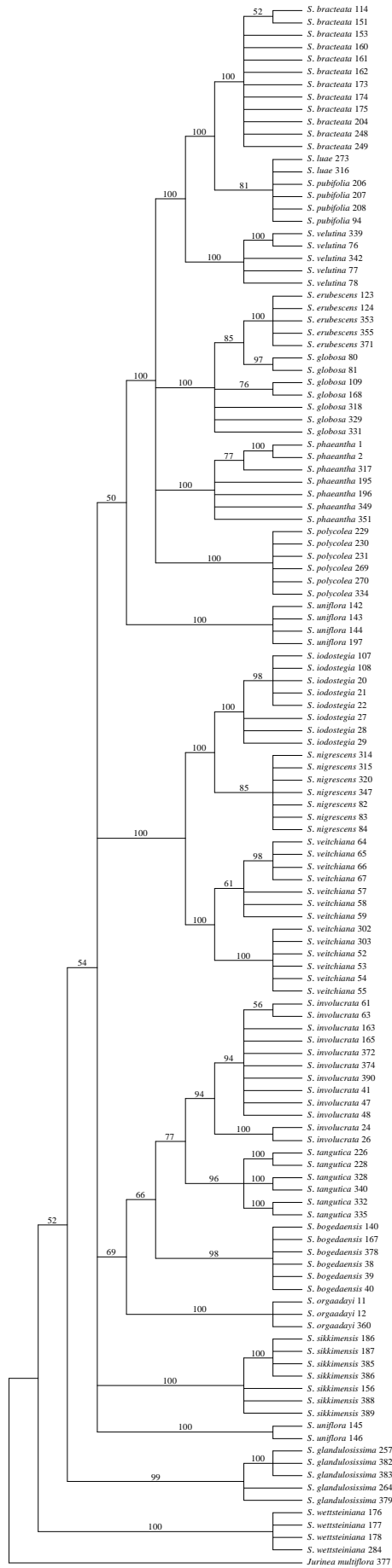

Supplement: Supplemental Information 1 [file peerj-07-6357-s001.zip › Supporting Information/Fig. S5.pdf]

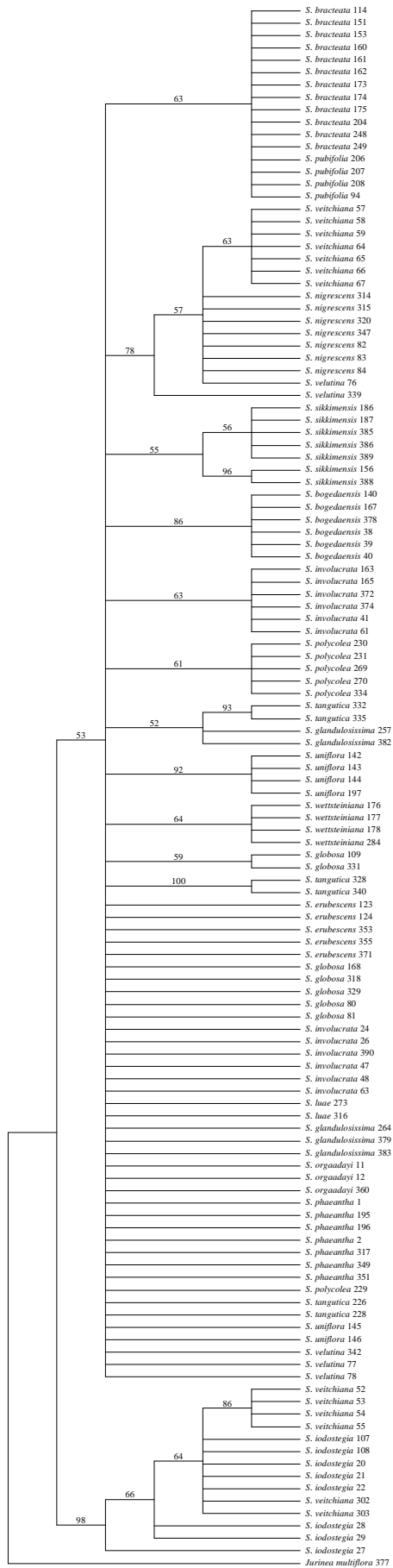

Supplement: Supplemental Information 1 [file peerj-07-6357-s001.zip › Supporting Information/Fig. S50.pdf]

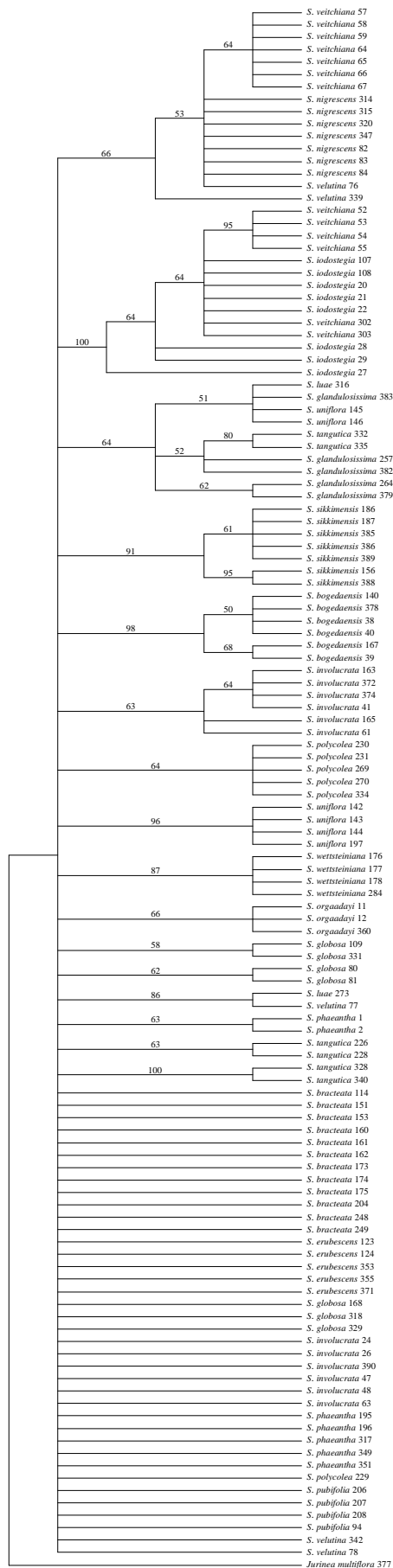

Supplement: Supplemental Information 1 [file peerj-07-6357-s001.zip › Supporting Information/Fig. S51.pdf]

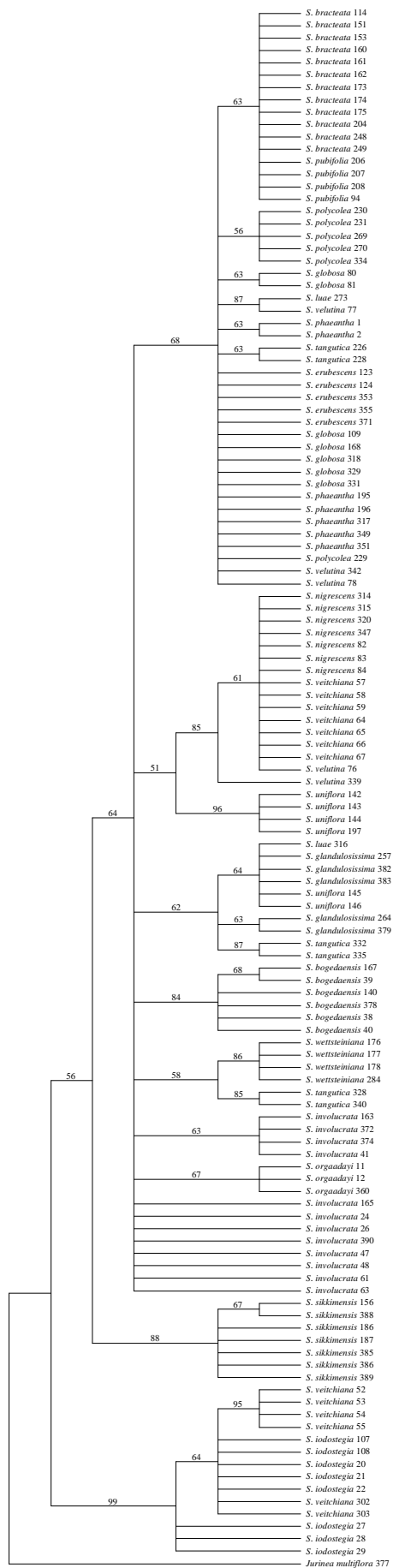

Supplement: Supplemental Information 1 [file peerj-07-6357-s001.zip › Supporting Information/Fig. S52.pdf]

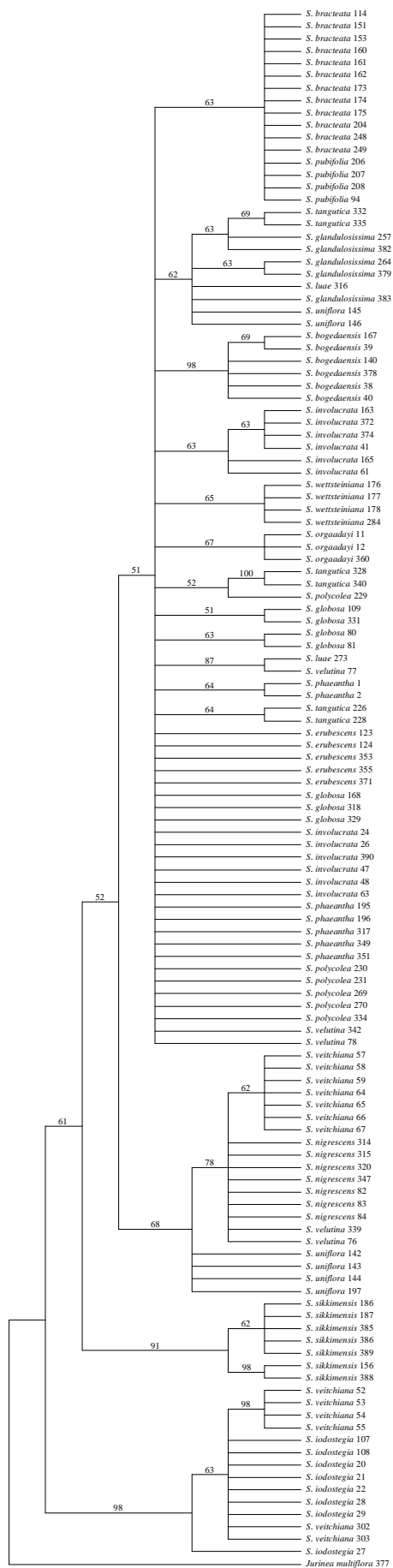

Supplement: Supplemental Information 1 [file peerj-07-6357-s001.zip › Supporting Information/Fig. S53.pdf]

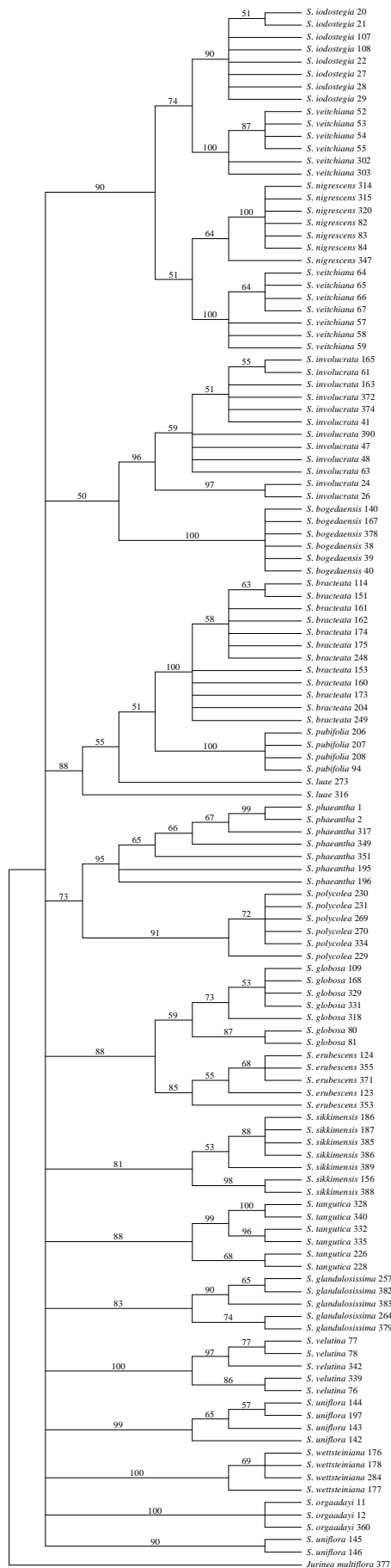

Supplement: Supplemental Information 1 [file peerj-07-6357-s001.zip › Supporting Information/Fig. S54.pdf]

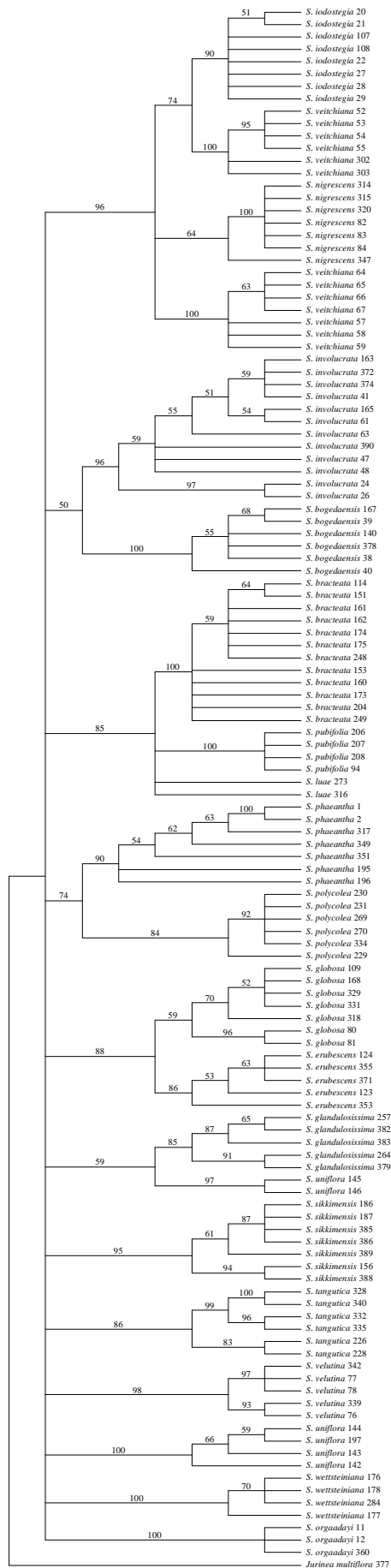

Supplement: Supplemental Information 1 [file peerj-07-6357-s001.zip › Supporting Information/Fig. S55.pdf]

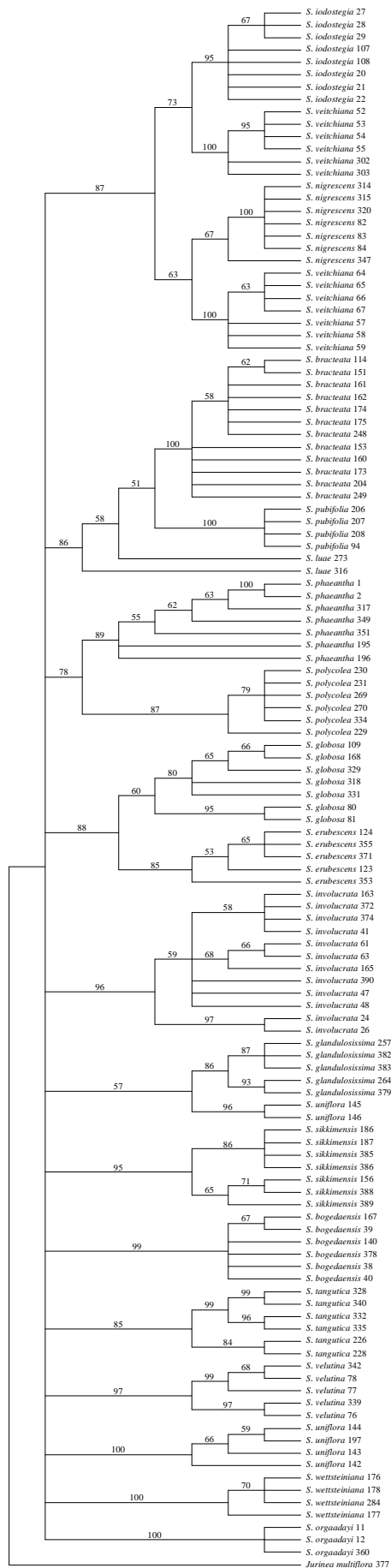

Supplement: Supplemental Information 1 [file peerj-07-6357-s001.zip › Supporting Information/Fig. S56.pdf]

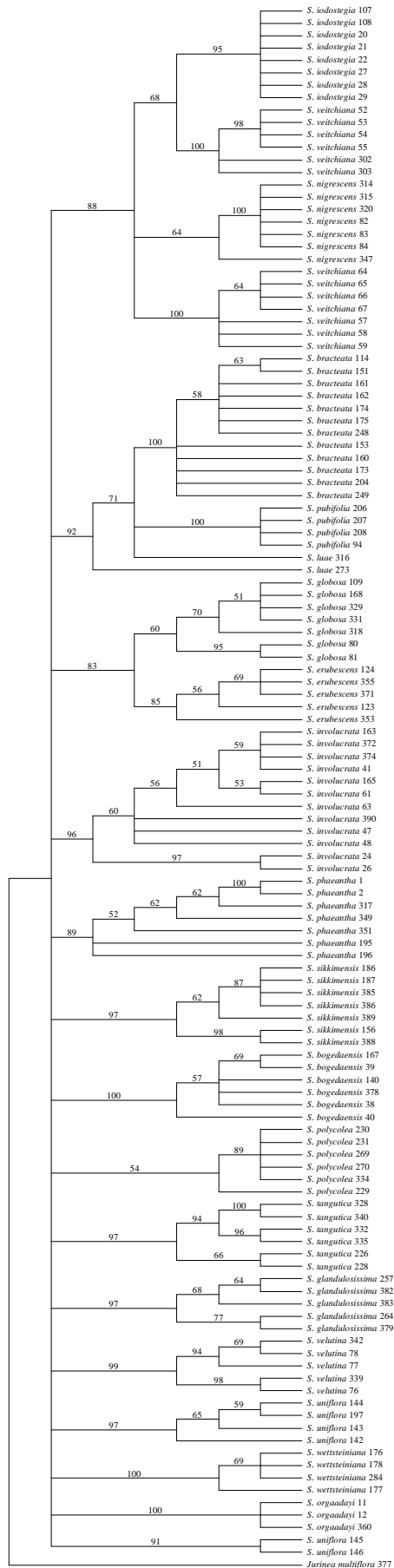

Supplement: Supplemental Information 1 [file peerj-07-6357-s001.zip › Supporting Information/Fig. S57.pdf]

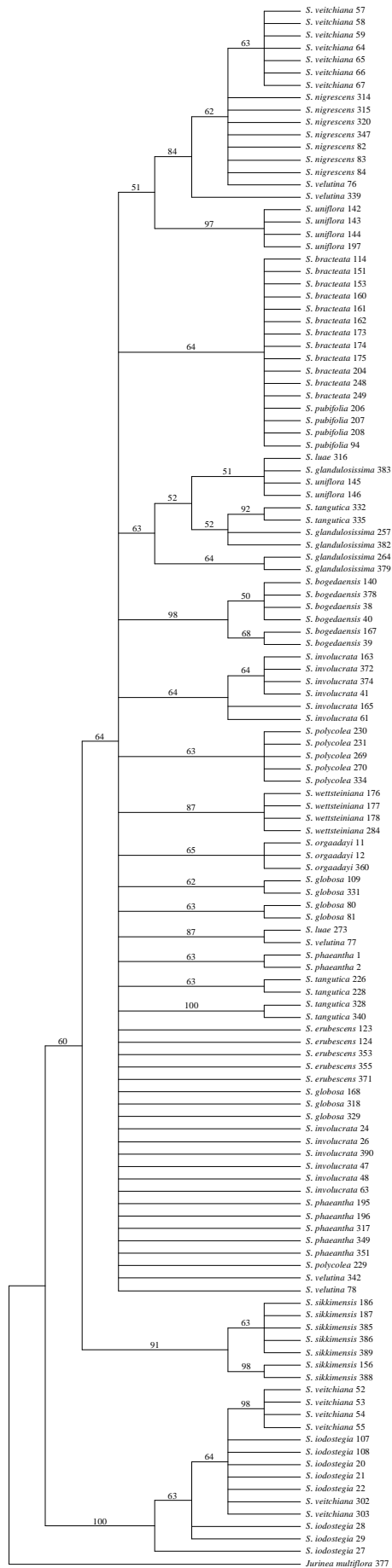

Supplement: Supplemental Information 1 [file peerj-07-6357-s001.zip › Supporting Information/Fig. S58.pdf]

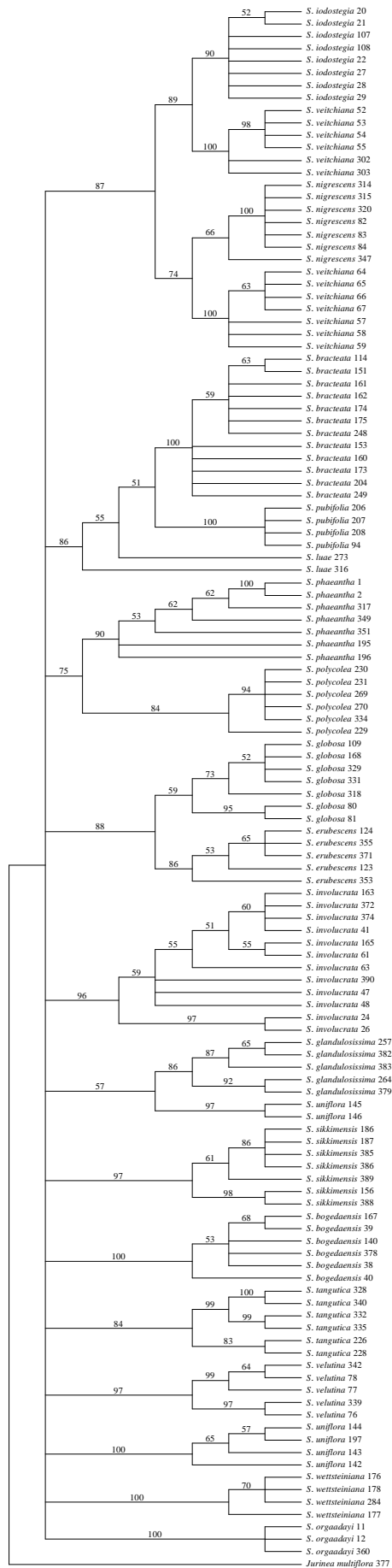

Supplement: Supplemental Information 1 [file peerj-07-6357-s001.zip › Supporting Information/Fig. S59.pdf]

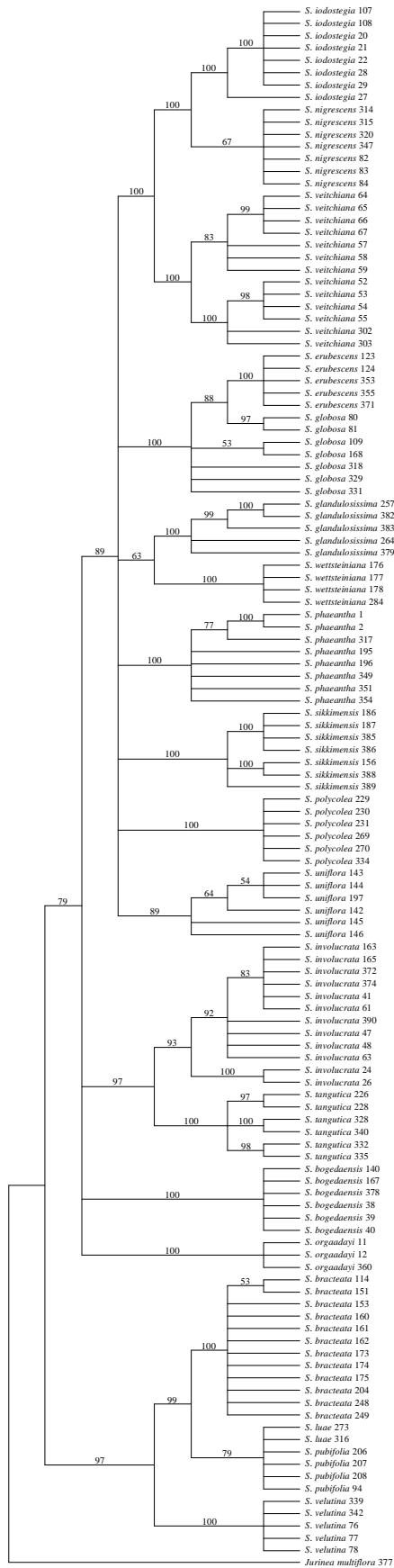

Supplement: Supplemental Information 1 [file peerj-07-6357-s001.zip › Supporting Information/Fig. S6.pdf]

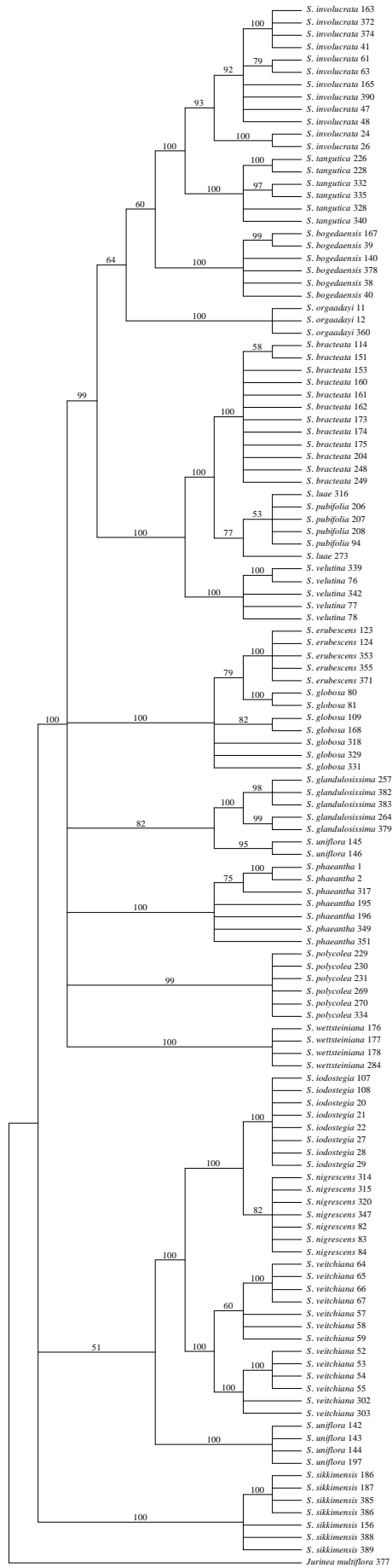

Supplement: Supplemental Information 1 [file peerj-07-6357-s001.zip › Supporting Information/Fig. S7.pdf]

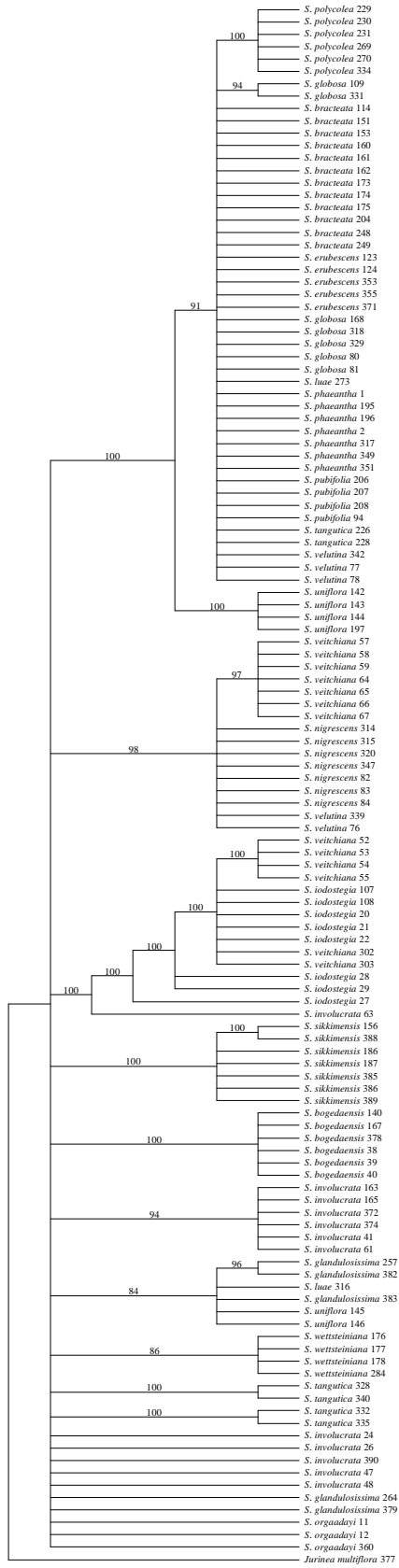

Supplement: Supplemental Information 1 [file peerj-07-6357-s001.zip › Supporting Information/Fig. S8.pdf]

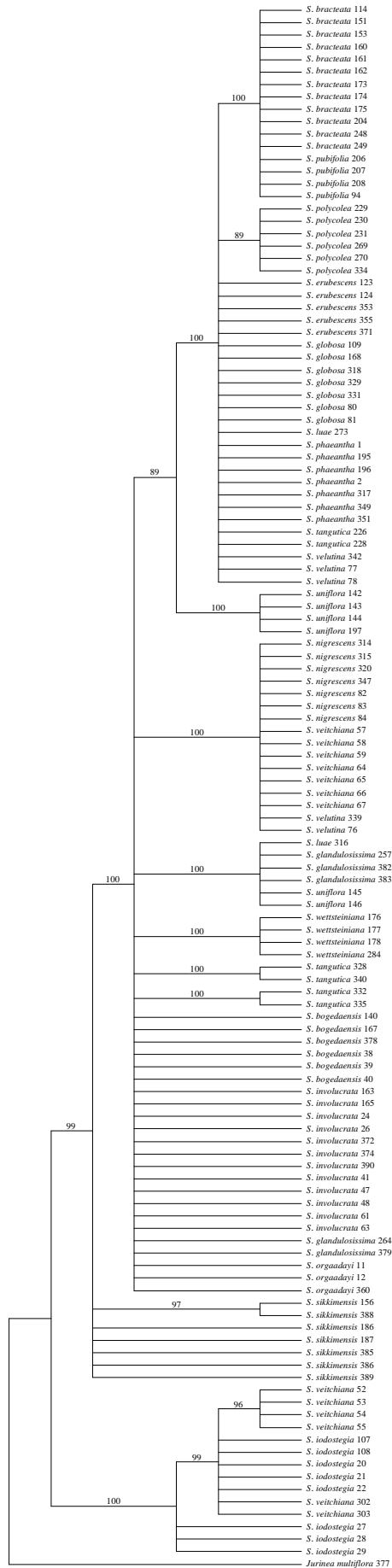

Supplement: Supplemental Information 1 [file peerj-07-6357-s001.zip › Supporting Information/Fig. S9.pdf]
